# Mathematical model and computational scheme for multi-phase modeling of cellular population and microenvironmental dynamics in soft tissue

**DOI:** 10.1371/journal.pone.0260108

**Published:** 2021-11-17

**Authors:** Gregory Baramidze, Victoria Baramidze, Ying Xu

**Affiliations:** 1 School of Computer Sciences, Western Illinois University, Macomb, Illinois, United States of America; 2 Department of Mathematics and Philosophy, Western Illinois University, Macomb, Illinois, United States of America; 3 Computational Systems Biology Lab, Department of Biochemistry and Molecular Biology, University of Georgia, Athens, Georgia, United States of America; University of New South Wales, AUSTRALIA

## Abstract

In this paper we introduce a system of partial differential equations that is capable of modeling a variety of dynamic processes in soft tissue cellular populations and their microenvironments. The model is designed to be general enough to simulate such processes as tissue regeneration, tumor growth, immune response, and many more. It also has built-in flexibility to include multiple chemical fields and/or sub-populations of cells, interstitial fluid and/or extracellular matrix. The model is derived from the conservation laws for mass and linear momentum and therefore can be classified as a continuum multi-phase model. A careful choice of state variables provides stability in solving the system of discretized equations defining advective flux terms. A concept of deviation from normal allows us to use simplified constitutive relations for stresses. We also present an algorithm for computing numerical approximations to the solutions of the system and discuss properties of these approximations. We demonstrate several examples of applications of the model. Numerical simulations show a significant potential of the model for simulating a variety of processes in soft tissues.

## Introduction

Numerous mathematical models and computational frameworks have been designed to represent complex dynamics in soft tissue with a significant portion of research revolving around modeling various processes in tumor development [1–11]. These models can also be used to simulate scenarios ranging from tissue oxygenation [12–18] to invasion [19–22] to tumor encapsulation [23, 24].

Most biological processes are interconnected, and complex relationships between them are often hard to understand and untangle, let alone quantify. Experimental investigations can be very time-consuming, expensive, and limited in scope. Experiments typically focus on a single point while keeping the other conditions fixed, making it hard to study systems level and dynamical behaviors. From this point of view, computational studies, in general, can be very advantageous, and computational models, in particular, can be used to elucidate various aspects of complex biological interactions and test the feasibility of different hypotheses on multiple levels.

The current work has the goal of creating a flexible computational framework that allows modelers to capture elements of mechanical and chemical process dynamics typical for spatially heterogeneous soft biological tissues, in which interactions between different types of cells and the micro-environment can be specified in variable detail (including interstitial fluid with various chemical agents, vascular networks, and extracellular matrix components). Target applications can include study of tissue regeneration, tumorigenesis, and cancer therapy, and others that share above mentioned characteristics.

Computational approaches to tumor growth modeling can be broadly subdivided into two main categories: discrete cells and continuum models (as well as the combination of both—hybrid models) [2, 5]. In the former, each individual cell is modeled as a discrete particle, represented by some computational entity (e.g., an object, or an agent), that can exhibit individual properties and reactions. In the latter, the cells are approximated as a continuous fluid, and partial-differential equations (PDEs) are used to describe their dynamics.

The discrete-cell approach allows integration of various properties into individual cells and observation of their dynamics separately. The approach is very flexible because it allows, at least theoretically, each individual cell to exhibit a unique phenotypical behavior. It can also allow incorporation of various biochemical processes within individual cells. This, of course, comes at a steep computational price—the more individual properties each cell can exhibit, the more computational power and time is needed. For a large system simulation, these computational issues can become prohibitively “expensive” [2].

Besides computational concerns, the discrete-cell approach may have some issues from a purely modeling point of view. A great number of details are needed to describe individual cells and their interactions with other cells and their environment, (for example, shapes and mechanical properties of cells, cell-cell and cell-ECM adhesion and interactions, details on cell motility and so on) while, at the same time, the biology related to these details is largely unclear or completely unknown. This means that modelers often have to fill in those knowledge gaps with guesses (although often biophysically driven) and simplifying assumptions, and this can often render the model unrealistic, thus devaluing the extra flexibility offered by the discrete-cell approach.

The continuum approach, in contrast, does not have individual cells and, instead, treats the tissue as a continuous deformable mass, utilizing the continuum mechanics tool set [5]. Rather than capturing characteristics of individual cells, these models introduce averaged cellular attributes. This allows handling larger systems and is, generally, less demanding on the computational resources. One of the trade-offs is a sharply decreased number of options for the control of heterogeneous properties of tumor cells. Not only is the individuality of cells within the model lost, but even the number of different types of cells (e.g., different mutated clones) becomes an “expensive” option—each new type has to be modeled by a separate PDE.

Since the continuum approach does not have a concept of individual cells, instead of counting the number of cells, the concept of *volume fraction* is often utilized for each cell type modeled by the system [5]. For most models, mass balance equations are used to formulate partial differential equations governing movement of these continuous fields of cells [25]. These equations may account for cell movement due to mechanical forces exerted on them by other components, random motility of cells (diffusion terms), and cell movement along existing gradients (chemotaxis and haptotaxis terms) [26].

In general, continuum models can be further subdivided into two categories: single phase and multiphase [5]. Single phase models treat tumor mass as a homogeneous component, sharply defined by its boundary. As a result, the equations describing the dynamics of the tumor’s growth are the equations for the boundary of the tumor. This approach does not provide framework for including the interactions of the multiple cell types and the extracellular matrix (ECM) into the model.

We chose a continuum multiphase approach, utilizing the concept of *volume fraction* for each of the multiple cell types modelled by the system [5]. In this approach, the governing equations apply to the entire domain and not just to the boundary, as opposed to the single phase models. This, in turn, allows for modeling interactions between different phases (components), including the ECM and interstitial fluid, which is why we ultimately settle on this strategy.

For the continuum based model, formulation begins with the set of advection equations, which can be derived directly from the mass balance principle. These equations contain *drift velocities* for each cell type, and the most difficult part is connecting these velocities to volume fractions and nutrient concentrations [25]. The process of completing the system of equations with additional relationships between the components, that will allow the system to become solvable, is often referred to as the *closure* of the system. Depending on the approach used to close the system, the models can acquire various characteristics in terms of the details they can capture, or the accuracy of their predictions.

We picked an approach that uses the momentum balance equations with constitutive relations, where the closure is based on the fundamental principle of conservation of linear momentum. (Two other commonly used approaches are using potential flow models and introducing an “action” function [25]). The resulting partial differential equations relate external forces acting on components to their stresses [5]. Particular forms of stresses vary from material to material, and several forms have been used in tissue modeling [23, 24, 27]. The external forces commonly include drag forces which are directly related to the relative velocities of the components. If one wishes to include such processes as random motility and/or chemotaxis, it is a straightforward task to incorporate additional force terms that give rise to these phenomena within the model.

Thus, we close the set of advection equations with momentum balance equations using drag forces and pressure to connect to components’ momentum transfers. Since we also aim to quantitatively take into account nutrient transport and signaling within tissue, the addition of the diffusion equations to model nutrients and microenvironment completes the set of differential equations defining the model.

The elements of our model pertaining to cell motion are closely related to the models presented in [23, 24]. There are, however, some important differences. We avoid making several simplifying assumptions included in these works. One of the most important ones is that we allow different components in our model to have different drag coefficients. Additionally, we avoid assuming spherical symmetry and solve the equations on a 2-dimensional grid, allowing more freedom of movement for the components in the model. For example, non-uniformity of the ECM and/or the spacing of the capillary sources in the domain may very well lead to the tumor shape variations reflecting local differences in micro environment and/or mechanical properties of the tissue, which can be captured by our model. We also slightly modify excess pressure functions and introduce an additional control over the ECM’s “active” response to changes in soft tissue. The approach developed in [23, 24] allows for multiple cell types (though the authors work with only three components, which suits their needs), and we take advantage of this by introducing multiple cell subpopulations. We also couple the equations governing growth and decay of the components with chemical fields.

Lastly, in order to translate our mathematical framework to computer simulation models, we develop a numerical treatment scheme that tries to balance accuracy with complexity and stability. Using a rectangular mesh we discretize space and time and apply a predictor-corrector method [28] to evolve the system. At every time step we solve the diffusion equations for chemical fields and momentum balance equations for system components to advance the mass conservation equations in time.

Some of the computational ideas are in line with the methods described by [9]. For example, we adapt the use of half-index locations, though we use a different time-dependent scheme. While the model in [9] assumes that at a given location all components of the system move with the same velocities, in contrast, we also allow each component to move at its own velocity, determined from the linear momentum balance equations. Additionally, we include interstitial fluid and the ECM components to represent more realistic interactions between the cells and the micro-environment.

From a computational point of view, one of the distinguishing characteristics of our approach is choosing advective flux terms as state variables, instead of velocities. This approach allows us to ensure that the linear system of equations resulting from the balance of linear momentum is invertable.

The paper is organized as follows: the mathematical formulation of the model is developed in Mathematical Model Development. Simulations and Experimental Results is devoted to several numerical examples demonstrating the capacity of the model to simulate soft tissue processes. We summarized the key characteristics of the model in Conclusions. The details of the numerical methods used to compute the approximations to the solutions of the model are presented in S1–S4 Appendices.

## Mathematical Model Development

The development of this model can be conceptually subdivided into three parts:

- A mathematical model of the population birth/death and movement dynamics for all of the components of the system.
- A mathematical model of the chemical species dynamics within the interstitial fluid (coupled with the first part)—the micro-environment.
- A numerical scheme that would translate/approximate the two previous parts into a form suitable for computer simulations.

The first two are addressed in the following subsections, while the bulk of the details for the numerical schema is presented in S1–S4 Appendices.

### System components dynamics

Consider a biological system, which is a sample of soft tissue, consisting of cells, interstitial fluid, and the extracellular matrix (ECM). We’ll refer to the different types of cells, together with the ECM and interstitial fluid as *components of the system*, and use *u*_*i*_ to denote a volume fraction of the *i*-th component, *i* = 0, ⋯, *n* + 1. (By volume fraction *u*_*i*_, we mean the fraction of the infinitesimal volume element occupied by the *i*-th component.) For convenience, we reserve index 0 for interstitial fluid and index *n* + 1 for the extracellular matrix, when present.

The system of partial differential equations governing our computational model is built upon several fundamental assumptions and concepts stemming from continuum mechanics and used by many researchers from the tumor dynamics modeling community (see, for example, [5, 9, 23–25, 29]):

1. Due to the conservation of mass, the volume fractions of the system’s components, *u*_*i*_-s, *i* = 0, ⋯, *n* + 1, each obey the advection equation
  $$\begin{array}{c} \frac{\partial u_{i}}{\partial t}+\nabla \cdot \left(u_{i}v_{i}\right)=g_{i}, \end{array}$$ (1)
  where **v**_*i*_ denotes the components’ velocity and *g*_*i*_ is its net production rate. The second term on the left-hand side of the equation, ∇ · (*u*_*i*_ **v**_*i*_), essentially means mass transport.
2. No net volume change occurs (i.e., everything is produced from something already in the system) and thus
  $$\begin{array}{c} \sum_{i=0}^{n+1}g_{i}=0; \end{array}$$ (2)
3. There are no voids in the system (or we can think of it as if all voids are filled with interstitial fluid) and thus
  $$\begin{array}{c} \sum_{i=0}^{n+1}u_{i}=1; \end{array}$$ (3)
4. Ignoring inertial effects (which is reasonable with a very slowly moving mass) and external forces (such as gravity), conservation of linear momentum implies that each component of the system obeys
  $$\begin{array}{c} \nabla \cdot \left(u_{i}\sigma_{i}\right)+F_{i}=0, \end{array}$$ (4)
  where ***σ***_*i*_ denotes the Cauchy stress of the component *i* and **F**_*i*_ is a superposition of forces that other components exert on the component *i*, *i* = 0, ⋯, *n* + 1. (One can think of stress as an internal force acting on the component as a reaction to an external force. It is a reflection of the mechanical properties of a component.).

Eqs (2) and (3), applied to the sum of the Eqs in (1), lead to the incompressibility condition

$$\begin{array}{c} \nabla \cdot \left(\sum_{i=0}^{n+1}u_{i}v_{i}\right)=0, \end{array}$$ (5)

which is used to complement Eq (4). This equation has a *stabilizing effect* on the model: at every moment of time and at every location of the domain it allows us to insist on a zero net mass transfer.

While some modelers restrict their attention to cellular components only [9], others include interstitial fluid, as well as the extracellular matrix [23, 24]. The net production rates of the components may depend on volume fractions of other components, as well as various chemical concentrations when such are included and coupled with the above mentioned equations. Defining these rates does not usually present a challenge, and solving Eq (1) can be easily accomplished with a variety of numerical methods, as long as the velocities **v**_*i*_ of the components are available.

If we use the conservation of the linear momentum, the equations for obtaining the velocities **v**_*i*_ depend on the choices of constitutive relations defining the Cauchy stresses and the interactions between various components of the system captured by the model’s force terms in (4). The superposition of forces acting on the component *i* consists of the net static pressure, *p*∇*u*_*i*_, exerted on the component *i*, with *p* denoting so-called interphase pressure and the frictional drag due to the relative motion of the components [23, 24, 29]:

$$\begin{array}{c} F_{i}=p\nabla u_{i}+\sum_{j=0,j\neq i}^{n+1}{\hat{\alpha }}_{ij}u_{i}u_{j}\left(v_{j}-v_{i}\right),\;\;i=0,\cdots ,n+1, \end{array}$$ (6)

where ${\hat{\alpha }}_{ij}$ is the drag coefficient for the frictional force between the components *i* and *j*. (Alternatively, one can use an approach where the velocities are computed as proportional to the gradient of pressure [9]. This simplifies the model but the components lose the ability to move independently—they all end up moving in the same direction.).

Back in (4), the Cauchy stress ***σ***_*i*_ varies from model to model and is defined based on the assumptions of mechanical properties of the corresponding component *i*. For example [23, 29], assume that components in their models behave as incompressible isotropic Newtonian fluids with constant viscosity across the domain, and as such obey

$$\begin{array}{c} \sigma_{i}=-q_{i}I+\lambda_{i}tr\left(E_{i}\right)I+2\mu_{i}E_{i},\;\;i=0,\cdots ,n. \end{array}$$ (7)

Here, $E_{i}=1/2(\nabla v_{i}+\nabla v_{i}^{T})$ is the rate of strain tensor of component *i*, **I** is the identity tensor, *q*_*i*_ is the macroscopic intra-phase pressure in the component *i*, *μ*_*i*_ is the sheer viscosity of the component, and λ_*i*_ is a coefficient characterizing the dilation viscosity of the component *i*.

In [23], the following form of stress-strain relationship is proposed for all components (phases):

$$\begin{array}{c} \sigma_{i}=\left(-p-\Psi_{i}\right)I, \end{array}$$ (8)

where Ψ_*i*_ represents an additional isotropic pressure that distinguishes the component *i* from other components, and Ψ_0_ = 0.

We propose to define the Cauchy stress by

$$\begin{array}{c} \sigma_{i}=-pI-\Psi_{i} \end{array}$$ (9)

for all of our components, where **Ψ**_*i*_ is a general tensor meant to capture distinguishing mechanical characteristics of the component *i*. We also assume that **Ψ**_0_ = 0. This formulation combines the notions of Cauchy stress given in (7) and (8). It also allows us to carry out the derivation of the final equations for the model without specifying the stress-strain constitutive relationships just yet. This way the constitutive stress-strain relations are decided on when one is ready to apply the model to a particular biological problem at hand.

Using (6) and (9) in (4) we obtain

$$\begin{array}{c} -u_{i}\nabla p-\nabla \cdot \left(u_{i}\Psi_{i}\right)+\sum_{j=0,j\neq i}^{n+1}{\hat{\alpha }}_{ij}u_{i}u_{j}\left(v_{j}-v_{i}\right)=0,\;\;i=0,...,n+1. \end{array}$$ (10)

Suppose now that the tensors **Ψ**_*i*_ are defined in terms of other variables or known quantities in our model and that we know the volume fractions *u*_*i*_, *i* = 0, ⋯, *n* + 1 at some moment of time *t*. We plan to use the *n* + 2 vector Eq (10) together with a scalar incompressibility condition (5) to compute the velocities **v**_*i*_, *i* = 0, ⋯, *n* + 1 and the interphase pressure *p*. However, while formally the number of equations is equal to the number of unknowns, our system is not well defined. This is due to the fact that the gradient of the interphase pressure can be excluded from the system of Eq (10), as we show below, in which case we end up needing additional restrictions to find a unique solution.

Indeed, consider adding Eq (10) for all *i* = 0, ⋯, *n* + 1. As drag forces cancel out, we get

$$\nabla p=-\nabla \cdot \sum_{j=0}^{n+1}u_{j}\Psi_{j}=-\nabla \cdot \sum_{j=1}^{n+1}u_{j}\Psi_{j},$$

and thus ∇*p* can be eliminated from Eq (10) entirely, resulting in

$$\begin{array}{c} u_{i}\nabla \cdot \sum_{j=1}^{n+1}u_{j}\Psi_{j}-\nabla \cdot \left(u_{i}\Psi_{i}\right)+\sum_{j=0,j\neq i}^{n+1}{\hat{\alpha }}_{ij}u_{i}u_{j}\left(v_{j}-v_{i}\right)=0,\;\;i=0,...,n+1, \end{array}$$ (11)

which, together with (5), are not linearly independent anymore.

To resolve this issue, we proceed by eliminating **v**_0_ from the system of Eq (11) and dropping the equation for *i* = 0 from the set (11).

Note that

$$\begin{array}{c} \sum_{i=0}^{n+1}u_{i}v_{i}=0 \end{array}$$ (12)

is the trivial solution of (5), which leads to

$$\begin{array}{c} u_{0}v_{0}=-\sum_{j=1}^{n+1}u_{j}v_{j} \end{array}$$ (13)

and allows us to eliminate *v*_0_ **v**_0_ from (11). As a result, Eq (11) are simplified to

$$\begin{array}{c} ({\hat{\alpha }}_{i0}u_{i}+\sum_{j=0,j\neq i}^{n+1}{\hat{\alpha }}_{ij}u_{j})u_{i}v_{i}+u_{i}\sum_{j=1,j\neq i}^{n+1}({\hat{\alpha }}_{i0}-{\hat{\alpha }}_{ij})u_{j}v_{j}=\\ =u_{i}\nabla \cdot \sum_{j=1}^{n+1}u_{j}\Psi_{j}-\nabla \cdot \left(u_{i}\Psi_{i}\right),\;\;i=1,...,n+1. \end{array}$$ (14)

From this point on, we shall assume that all of **Ψ**_*i*_’s are defined and independent of velocities (as in [23]). It is important to note that in this case the system (14) is linear with respect to **v**_*i*_’s. This becomes crucial later on, when numerically computing velocities in practice.

At this stage, as long as **Ψ**_*i*_’s are defined in terms of known quantities and previously introduced variables, the combined system of Eqs (1) and (14) completely defines the variables *u*_*i*_ and **v**_*i*_, *i* = 1, ⋯, *n* + 1, with (3) and (13) used to compute the volume fraction and the velocity of the interstitial fluid whenever needed.

It now comes down to the selection of the form of **Ψ**_*i*_’s to be used in (14). The choice depends on the complexities of the mechanical responses needed to be reflected by the model. In the examples presented in this paper we introduce a simple form

$$\begin{array}{c} \Psi_{i}=k_{i}{\left(u_{0}^{opt}-u_{0}\right)}_{+}I=:\Psi_{i}I,\;\;i=1,\cdots ,n+1. \end{array}$$ (15)

Here, $u_{0}^{opt}$ is a parameter representing the optimal or typical volume fraction of the interstitial fluid, which may or may not be constant over the domain. Whenever the current value of the volume fraction of the interstitial fluid deviates from the optimal, the tissue is considered to be under stress. The magnitude of the effect of the stress on the *i*-th component of the system is controlled by the *strain-stress coefficient* *k*_*i*_.

Since Ψ_*i*_’s defined above are scalars, Eq (14) simplify as

$$\begin{array}{c} ({\hat{\alpha }}_{i0}u_{i}+\sum_{j=0,j\neq i}^{n+1}{\hat{\alpha }}_{ij}u_{j})u_{i}v_{i}+u_{i}\sum_{j=1,j\neq i}^{n+1}({\hat{\alpha }}_{i0}-{\hat{\alpha }}_{ij})u_{j}v_{j}\\ =u_{i}\nabla \sum_{j=1}^{n+1}u_{j}\Psi_{j}-\nabla \left(u_{i}\Psi_{i}\right),\;\;i=1,...,n+1. \end{array}$$ (16)

In contrast to [23, 24, 29] this approach does not assume that all the drag coefficients are equal, and unlike [23, 24] it also does not assume radial symmetry.

An additional critical observation: if a component *u*_*i*_ at some location in a sample has zero volume fraction, its velocity cannot be determined from (16). This causes problems when the equations are discretized—the coefficient matrix becomes singular. A way around this problem is to treat *u*_*i*_ **v**_*i*_ as state variables instead of just **v**_*i*_. Then, as we show in S3 Appendix subject to some conditions on the drag coefficients, the system of equations for determining *u*_*i*_ **v**_*i*_ over a grid has a unique solution.

### Oxygen and glucose concentrations

In this section we describe chemical components that may be included in our model to represent the influence of micro-environment on the cells, as well as the impact of the cells’ metabolisms on the micro-environment. These relationships between cells and micro-environmental factors allow us to highlight both direct and indirect interactions between cells, the extracellular matrix, and the interstitial fluid, and offer meaningful conclusions based on the results of the numerical simulations of the model.

Two main chemicals we are interested in are oxygen and glucose, both delivered to tissues by the vascular network. Adding *H*^+^ (to capture acidity), *VEGF*’s (blood vessels growth factors), or any other chemical fields into the mix would be done in a similar manner.

Assuming that the chemicals diffuse in the interstitial fluid, let *o*_*i*_ denote the fraction of the interstitial fluid occupied by the *i*-th chemical, *i* = 1, …, *m*. Then *u*_0_ *o*_*i*_ represents the actual volume fraction of the *i*-th chemical. This volume fraction obeys the diffusion-advection equation

$$\frac{\partial (u_{0}o_{i})}{\partial t}+\nabla \cdot \left(u_{0}o_{i}v_{i}\right)=r_{i}-c_{i},$$

where **v**_*i*_ is the velocity of the species, *r*_*i*_ is a production rate and *c*_*i*_ is a consumption rate of *u*_0_ *o*_*i*_. The velocity **v**_*i*_ can be viewed as a superposition of the velocity of the interstitial fluid **v**_0_ and the velocity of the chemical species relative to the interstitial fluid, **v**_*i*0_ (in other words, the diffusive velocity of the chemical *i*), that is **v**_*i*_ = **v**_0_ + **v**_*i*0_. Then the above equation can be written as

$$\frac{\partial (u_{0}o_{i})}{\partial t}+\nabla \cdot \left(o_{i}\left(u_{0}v_{0}\right)\right)+\nabla \cdot \left(u_{0}o_{i}v_{i0}\right)=r_{i}-c_{i}.$$

Using Fick’s law (see [10], for example), *u*_0_ *o*_*i*_ **v**_*i*0_ = −*D*_*i*_ ∇*o*_*i*_, where *D*_*i*_ is the diffusion coefficient, Eq (1) for *u*_0_, and product rule for differentiation, we get

$$u_{0}\frac{\partial o_{i}}{\partial t}+o_{i}\left(g_{0}-\nabla \cdot \left(u_{0}v_{0}\right)\right)+\nabla \cdot \left(o_{i}\left(u_{0}v_{0}\right)\right)-\nabla \cdot \left(D_{i}\nabla o_{i}\right)=r_{i}-c_{i}.$$

Simplifying this equation and also noting that we are interested in a steady-state solution of the equation (since the diffusion of chemicals is a much quicker process than that of cell movement and growth), we obtain

$$\begin{array}{c} -D_{i}\Delta o_{i}+\nabla o_{i}\cdot \left(u_{0}v_{0}-\nabla D_{i}\right)+o_{i}g_{0}-r_{i}+c_{i}=0. \end{array}$$ (17)

Recall that *g*_0_, the rate of change of the interstitial fluid due to growth and decay of other components in the system, can be described by

$$g_{0}=-\sum_{i=1}^{n+1}g_{i},$$

since $\sum_{i=0}^{n+1}g_{i}=0$. The functions *g*_*i*_ depend on birth-death rates of the present cells, as well as volume fraction changes of the extracellular matrix, which in turn might depend on the current values of the chemical species. Thus *g*_0_ = *g*_0_(*o*_*i*_), making Eq (17) non-linear with respect to *o*_*i*_.

In [10] it is suggested that the diffusion coefficient varies over the computational domain with the availability of the interstitial fluid. More specifically, $D_{i}={\hat{D}}_{i}u_{0}^{\delta }$ for some constant parameters ${\hat{D}}_{i}>0$ and *δ* > 1. Eq (17) then becomes

$$\begin{array}{c} -{\hat{D}}_{i}u_{0}^{\delta }\Delta o_{i}+\nabla o_{i}\cdot \left(u_{0}v_{0}-{\hat{D}}_{i}\nabla u_{0}^{\delta }\right)+o_{i}g_{0}-r_{i}+c_{i}=0. \end{array}$$ (18)

The consumption-production terms *r*_*i*_ and *c*_*i*_ vary from species to species and may depend on the presence of other components of the model. The consumption *c*_*i*_ can be assumed to be of the form

$$\begin{array}{c} c_{i}=o_{i}u_{0}\left(\sum_{j=1}^{n+1}c_{ij}u_{j}+\lambda \right), \end{array}$$ (19)

where *c*_*ij*_ is the rate of consumption of the chemical *i* by the component *j*, and λ is the rate of natural decay of *o*_*i*_. In general, the rates of consumption *c*_*ij*_ may depend on the micro-environment, therefore *c*_*i*_ may add a nonlinear component to the diffusion equation.

The rate of production of chemical *i*, *r*_*i*_, depends on several factors and varies from chemical to chemical. For example, oxygen and glucose are produced by capillary sources, while lactate is produced by tumor cells when fermentation is in place.

Thus, in case of lactate, we have to specify *r*_*i*_, while for oxygen and glucose, it is reasonable to solve

$$\begin{array}{c} -{\hat{D}}_{i}u_{0}^{\delta }\Delta o_{i}+\nabla o_{i}\cdot \left(u_{0}v_{0}-{\hat{D}}_{i}\nabla u_{0}^{\delta }\right)+o_{i}g_{0}+o_{i}u_{0}\sum_{j=1}^{n+1}c_{ij}u_{j}=0 \end{array}$$ (20)

in the computational domain Ω, subject to the following conditions on the boundary of Ω:

$$o_{i}{|}_{\partial \Omega }=\bar{o_{i}}$$

for some constant value $\bar{o_{i}}$, and, additional conditions,

$$\begin{array}{c} o_{i}\left(W\right)=\tilde{o_{i}}\left(W\right), \end{array}$$ (21)

where *W* denotes a set of capillary locations in Ω and $\tilde{o_{i}}\left(W\right)$ is a prescribed value of *o*_*i*_ at these locations. Oxygen partial pressure varies from vessel to vessel; a modeler may assign random values to the capillary sources or a single value for simplicity. It is also possible to change these values during simulations depending on the experimental design. For example, one can simulate fluctuating values at the capillary sources imitating cycling hypoxia and/or capillary damage or closure.

### Numerical solution

The final system of Eq (1) is solved numerically. We discretize space and time by parameters *h* and Δ*t*, respectively. Once the spacial derivatives involved are approximated by finite differences, the equations can be treated as a system of ordinary differential equations with respect to time. Then the numerical solutions are computed by a method based on a Predictor-Corrector scheme also classified as a 2-stage Runge-Kutta method. For the approach to work, before we are able to advance from time step *t*^(*ℓ*)^ to *t*^(*ℓ*+1)^ we have to compute the velocities of the components at the moment of time *t*^(*ℓ*)^ using Eq (16). Additionally, if in a given problem, the net production rates, *g*_*i*_, are influenced by chemical fields, we solve the diffusion Eq (18) for the relevant chemical species. To solve the latter PDEs, which are often nonlinear, their spatially discretized versions are solved by a combination of finite difference approach and Newton’s method.

We present the full details of the numerical scheme in S1–S4 Appendices.

## Simulations and Experimental Results

To illustrate the model’s ability to simulate various dynamic processes in soft tissue we set up a series of computational experiments based on a couple of simple scenarios. Our goal is not to conduct a detailed biological study, but to demonstrate the model’s potential for future research.

The following subsections deal with tissue regeneration phenomena with and without a vascular network explicitly present, and the ECM deformation during tumorigenesis. The model can be easily adopted to deal with more complex scenarios by straightforward inclusion of additional components and/or chemical species, varying constitutive relations, as well as introducing spacial heterogeneity within a tissue sample.

Finally, we conclude this section with experiments that demonstrate model accuracy and predictive power.

### Tissue regeneration

Multiple models for wound healing and tissue regeneration have been proposed over the years. As we review a few examples below, we note that while our model has some features in common with what’s been proposed in literature, it also has several distinct characteristics.

In [30], authors hypothesise that a single chemical with a simple regulatory effect can account for the healing of circular epidermal wounds. The system of equations modeling the process consists of an equation for cell density similar to (1), and an equation for the chemical density similar to (20). Cell motion other than diffusion is not reflected in the model.

In [31], skin wound healing is modeled by a cell-ECM system coupled with two chemicals, defined as activator and inhibitor. The equations describing the components are similar to Eq (1), except that the mass transfer terms have explicit forms limited to specific processes, such as diffusion and chemotaxis. Additionally, the system does not benefit from the inclusion of Eqs (3) and (5).

In [32], a four component model is proposed to account for two fundamental processes involved in the repair of an acute dermal wound: inflammatory response and fibroplasia. Each of the four PDE’s can be seen as a special case of Eq (1). This system does not include Eqs (3) and (5) either and has specific definitions for mass transfer terms.

In [33], the authors present a complex model for chronic wounds, with the equations defining the behavior of the components similar to (1). The authors assume that all components of the model move with the same velocity.

As seen above, in many cases, modelers resolve the difficulty of defining velocities of the model’s components by postulating explicit formulations of the components’ motion. In contrast, our model relies on conservation of momentum, which allows us to determine these velocities by accounting for local forces, subject to the incompressibility condition. These principles have a stabilizing effect on the solutions of the systems of PDEs governing the dynamics of the model.

In what follows, we first set up a simple model of tissue regeneration with just three components: interstitial fluid, live cells, and the extracellular matrix. In the midst of a 2D grid of live cells we introduce an irregularly shaped “wound” (a region without cells) and let the surrounding cells “grow into” the wound. The model represents the system in which the cells can divide and grow, depending on the irregularities of the tissue, the ECM has the ability to regenerate (as if secreted from cells) when damaged, and both components can move due to internal stresses and are subject to frictional forces.

Denote *u*_0_ to be the volume fraction of interstitial fluid, *u*_1_—live cells, *u*_2_—the extracellular matrix. By (1), our system obeys:

$$\begin{array}{c} \frac{\partial u_{i}}{\partial t}+\nabla \cdot \left(u_{i}v_{i}\right)=g_{i},\;\;i=1,2, \end{array}$$

and by (3), *u*_0_ = 1 − *u*_1_ − *u*_2_. By the force balance Eq (16), *u*_1_ **v**_1_ and *u*_2_ **v**_2_ obey

$$\begin{array}{c} ({\hat{\alpha }}_{i0}u_{i}+\sum_{j=0,j\neq i}^{n+1}{\hat{\alpha }}_{ij}u_{j})u_{i}v_{i}+u_{i}\sum_{j=1,j\neq i}^{n+1}({\hat{\alpha }}_{i0}-{\hat{\alpha }}_{ij})u_{j}v_{j}\\ =\nabla \left(u_{i}\Psi_{i}\right)-u_{i}\nabla \sum_{j=1}^{n+1}u_{j}\Psi_{j},\;\;i=1,2, \end{array}$$

where, using the form we introduced in (15), the stresses are defined by

$$\Psi_{i}=k_{i}{\left(u_{0}^{opt}-u_{0}\right)}_{+}\;,\;\;\;i=1,2.$$

For simplicity, we assume that the drag coefficients, ${\hat{\alpha }}_{ij}$, are the same for all pairs of components. Recall also that by (13),

$$\begin{array}{c} u_{0}v_{0}=-u_{1}v_{1}-u_{2}v_{2}. \end{array}$$

To complete the model, we now define constitutive relations for the cellular and the ECM components’ net production rates *g*_1_ and *g*_2_ (*g*_0_ = −*g*_1_ − *g*_2_ by Eq (2)).

In this example, we commonly assume that the rate of growth and the rate of decay of the cellular component is proportional to its volume fraction. This is not the case for the ECM’s growth rate, since it’s produced by cells and not the ECM itself:

$$g_{1}=\left(b_{1}-c_{1}\right)u_{1},\;\;\;\;\;\;\;\;\;\;\;g_{2}=b_{2}-d_{2}u_{2}.$$

Here, *b*_1_ *u*_1_ is the growth and *d*_1_ *u*_1_ is the decay rate of the cellular component, and *b*_2_, *d*_2_ *u*_2_ are the corresponding rates for the ECM. In this simple example we assume that no additional damage is occurring during the simulation, so *d*_1_ = *d*_2_ = 0.

For the cellular growth rate we define

$$b_{1}=b_{1,\text{max}}u_{0}{\left\|\nabla \left[{\left(u_{0}-u_{0}^{opt}\right)}_{+}\right]\right\|}_{2},$$

where *u*_0_ is included to indicate that the interstitial fluid is needed for cell division and growth, and *b*_1,max_ is a positive constant. The term $\|\nabla \left[{\left(u_{0}-u_{0}^{opt}\right)}_{+}\right]{\|}_{2}$ is a scalar measure of the gradient of the deviation from normal that triggers the cell division in the normal tissue (this is in contrast to uncontrolled cell division typical for tumor cells). The gradient above “senses” cellular volume fraction variations that are non uniform. This is what can be expected, for example, near the boundary of the wound.

The ECM growth rate

$$b_{2}=b_{2,\text{max}}u_{0}u_{1}{\left(u_{2}^{opt}-u_{2}\right)}_{+}$$

is defined to trigger the ECM regeneration when its volume fraction is below normal, specified by a parameter $u_{2}^{opt}$. The *u*_0_ term is present to indicate that the materials needed to build the ECM are delivered through the interstitial fluid and *b*_2,max_ is a positive constant. Additionally, *u*_1_ is present to indicate that the ECM is secreted by cells, and therefore its generation without presence of cells is impossible. These assumptions are not uncommon, for examples see [33, 34].

For simplicity, we assume that the tissue architecture is uniform, that is, the parameters $u_{0}^{opt}$ and $u_{2}^{opt}$ are constant throughout the domain.

**Fig 1:**
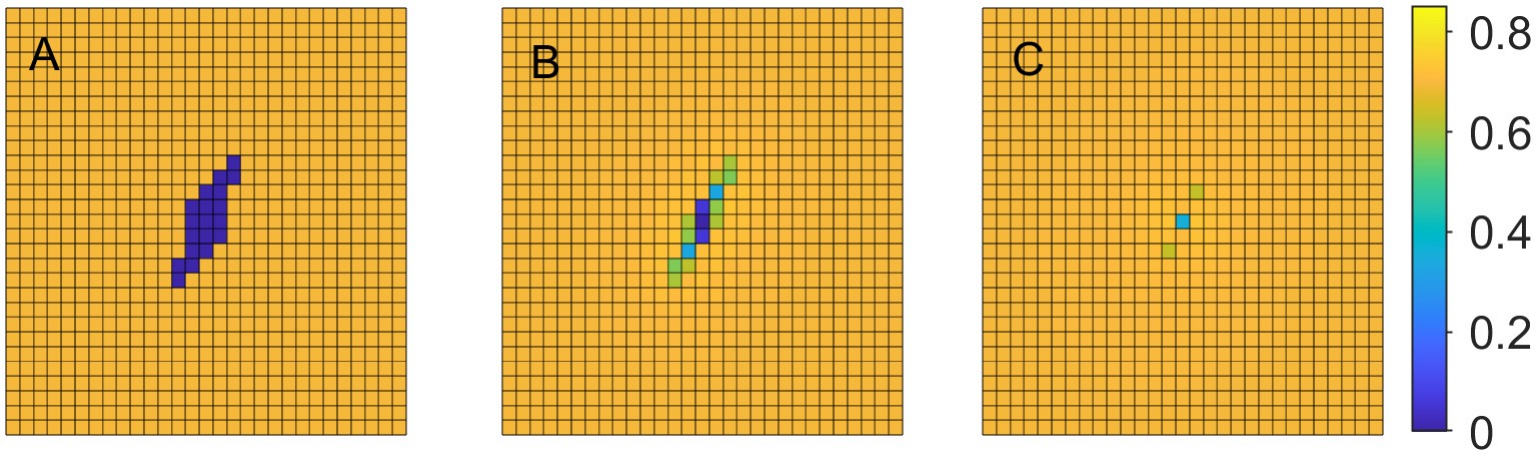
Tissue regeneration. Cellular volume fraction *u*_1_ at various time steps with *b*_1,max_ = 0.03. A: time step *t* = 1, B: time step *t* = 11, C: time step *t* = 21.

Initial conditions can be described as follows. A square domain with side length 0.3 mm is populated with the ECM, interstitial fluid and cells. The ECM volume fraction is 0.05 everywhere, the cellular volume fraction is

$$u_{1}\left(i,j\right)=\{\begin{array}{c} 0.7,(i,j)\in A,\\ 0,(i,j)\in B, \end{array}$$

where *B* is a set of locations in the domain representing an initial wound, and *A* is the set of all remaining locations. Fig 1A, illustrates this initial setup with *B* represented as blue and *A* as yellow squares.

Table 1 summarizes the parameter values that remain unchanged in the experiments of this example. Other parameters are varied to capture their influence on the processes simulated in the experiments and are specified as needed. Some parameter values were found in literature, others were estimated using the philosophy stated in [23]: to use the parameter values “for which the resulting simulations exhibit the desired qualitative behavior”. This often involves extensive numerical experimentation and qualitative and visual evaluation of the results.

**Table 1:** Parameter values for the experiments in tissue regeneration.

Parameter	Notation	Range	Units
Cell Growth Rate Coefficient	*b*_1,max_	0.03–0.05	*day* ^−1^
ECM Growth Rate Coefficient	*b*_2,max_	2–4	*day* ^−1^
Cell strain-stress coefficient	*k*_1_	300–500	*kg* *mm*^2^/*day*^2^
ECM strain-stress coefficient	*k*_2_	200–400	*kg* *mm*^2^/*day*^2^
Friction coefficients	${\hat{\alpha }}_{ij}$	10^5^ − 10^6^	*kg*/*day*
Optimal Interstitial Fluid	$u_{0}^{opt}$	0.25	–
Volume Fraction			–
Optimal ECM Volume Fraction	$u_{2}^{opt}$	0.05	–
Domain grid size	*h*	0.01	*mm*
Time step size	Δ*t*	0.25	*day*

To ensure the numerical stability of the simulation process (see S4 Appendix for details), the time step Δ*t* has to satisfy the condition (S4–1). Specifically, for the simulations that follow we require that

$$\Delta t\leq \frac{1}{b_{1,\text{max}}\left(1-u_{0}^{opt}\right)/h+b_{2,\text{max}}u_{2}^{opt}+\frac{4}{\hat{\alpha }}\left(k_{1}+k_{2}\right)u_{0}^{opt}/h^{2}}$$

The time step used in the simulations, Δ*t* = 0.25, satisfies the inequality above. Since this condition was derived for the worst case scenario throughout the process, in practice, often a larger time step may suffice.

**Example 1.a**. In the first example we set *b*_1,max_ = 0.03, *b*_2,max_ = 3 and *k*_1_ = 500, *k*_2_ = 300. Fig 1 demonstrates the changes in cellular volume fraction at the time steps 1, 11, and 21, where the changes from dark blue to lighter colors indicate cellular regeneration.

**Fig 2:**
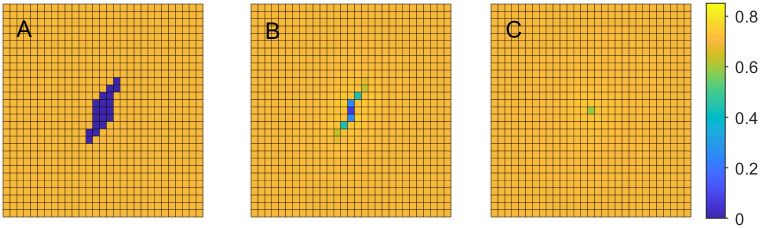
Tissue regeneration. Cellular volume fraction *u*_1_ at time steps *t* = 1, 11, 21 with *b*_1,max_ = 0.04. A: time step *t* = 1, B: time step *t* = 11, C: time step *t* = 21.

Similar experiments are carried out with *b*_1,max_ = 0.04 and 0.05. Fig 2 demonstrates the changes in cellular volume fraction at the same time steps as before, when *b*_1,max_ = 0.04. Comparing the Figs 1 and 2, we can observe that higher rate of cell division and growth leads to faster tissue regeneration.

**Fig 3:**
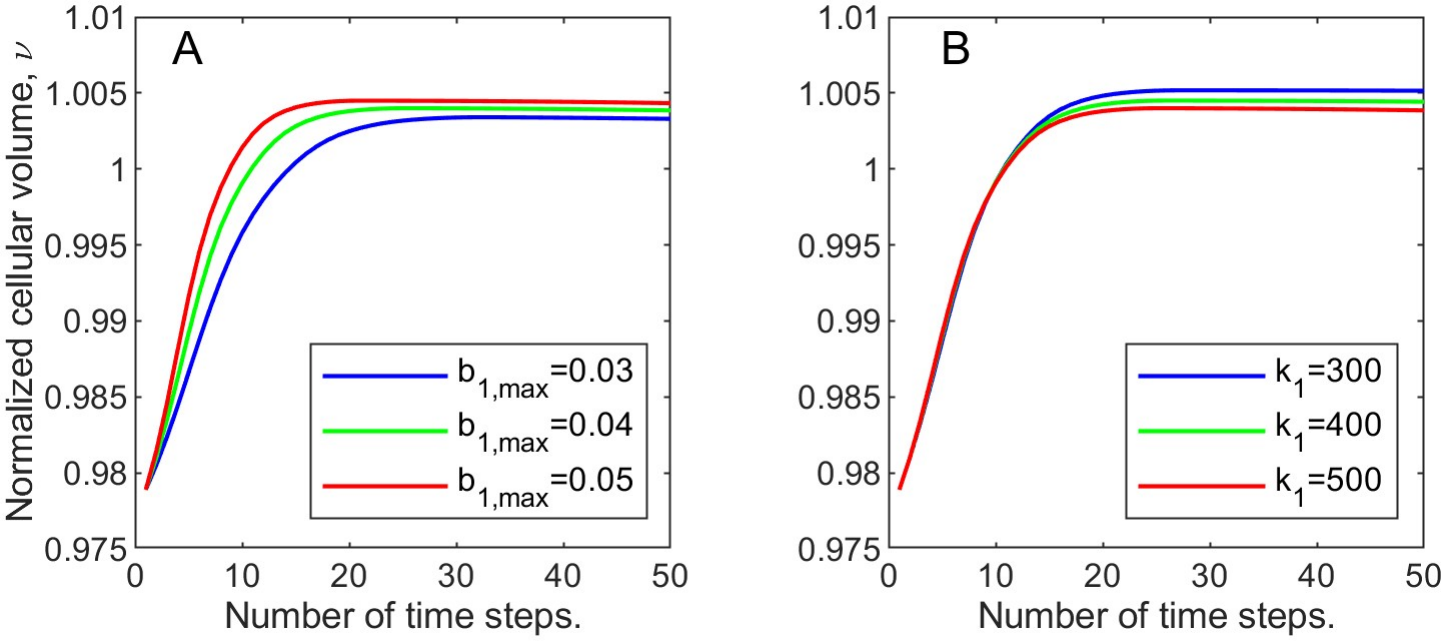
Normalized cellular volume, *ν*, changing with time. A: varying maximal cellular growth rate parameter, *b*_1,max_; B: varying strain parameter *k*_1_ for the cellular volume fraction.

To illustrate it even better, at every time step we compute the total cellular volume fraction *s* = ∑_*ij*_ *u*_1_(*i*, *j*) over the entire domain divided by the corresponding “pre-wound” value. We monitor this normalized cellular volume, *ν*, over time to track the change in the overall cell population for the three growth rates in Fig 3A (where the blue graph corresponds to the lowest and the red to the highest growth rates).

**Example 1.b**. In our next experiment we hold the growth rate coefficient *b*_1,max_ at 0.04 and vary the cell strain-stress parameter *k*_1_ from 300 to 500 in increments of 100. This parameter controls the amount of stress experienced by cells due to deviation from normal. In Fig 3B we recorded the normalized cellular volume values over time, for each value of *k*_1_. We can observe that the cellular volume recovery is the highest when *k*_1_ is the lowest.

**Fig 4:**
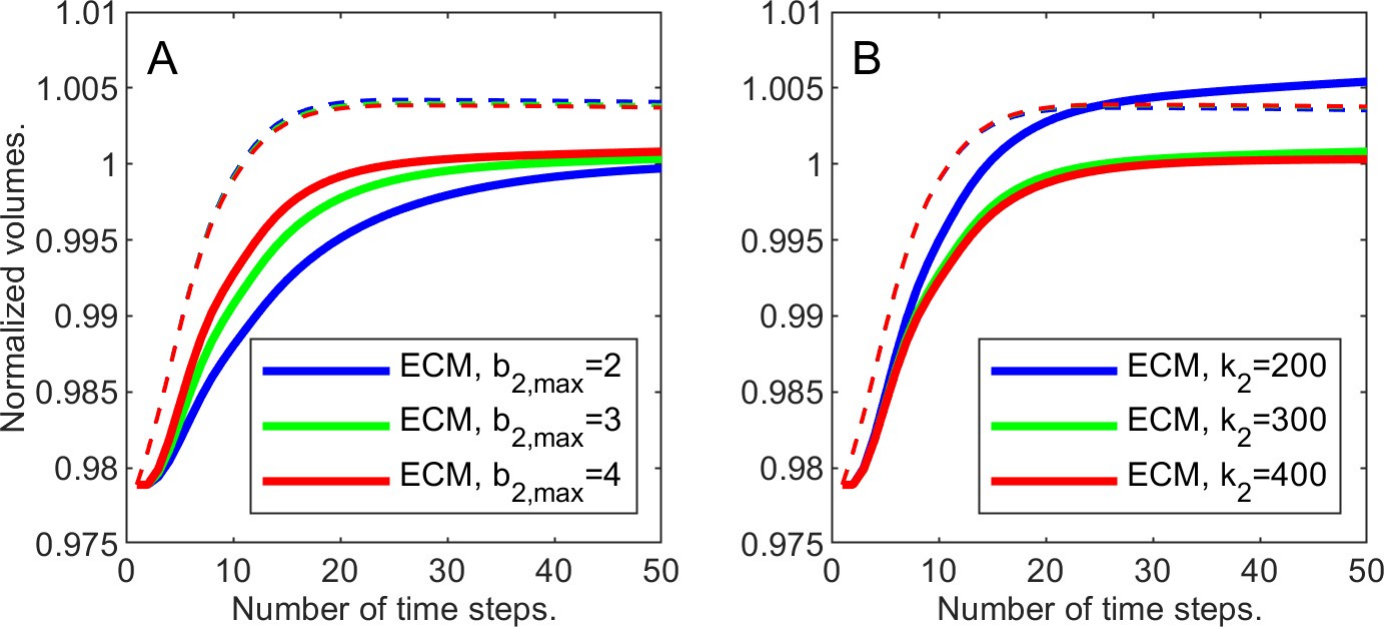
Cellular and ECM volume fractions changing with time. ECM—solid lines, cells—dashed lines. A: varying maximal rate of ECM growth, *b*_2,max_; B: Varying ECM strain parameter *k*_2_.

**Example 1.c**. In the above experiments we concentrated on cellular damage. The ECM volume fraction was set to the optimal value everywhere in the domain, including the “wound”, and its growth was not triggered. In the following simulations, we assume there is some initial ECM damage in addition to the cellular damage. Specifically,

$$u_{2}\left(i,j\right)=\{\begin{array}{c} 0.05,(i,j)\in A\\ 0,(i,j)\in B \end{array},$$

similar to *u*_1_. We keep *b*_1,max_ = 0.04 and *k*_1_ = 500, and vary *b*_2,max_ from 2 to 4, while keeping *k*_2_ = 300. Just like for the cellular volume fractions, at every time step we compute the normalized ECM volume, *μ*, that is the total ECM volume fraction ∑_*ij*_ *u*_2_(*i*, *j*) over the entire domain divided by the corresponding “pre-wound” value. Fig 4A demonstrates that the increase in the rate of regeneration leads to faster ECM recovery. Note also that, in all three cases, ECM regeneration lags behind the cellular regeneration, since we assumed that the ECM is secreted by cells.

**Example 1.d**. To complete this series of experiments, let us fix the ECM growth rate coefficient *b*_2,max_ at 4 and study the effects of changing the ECM strain-stress coefficient *k*_2_.

Consider Fig 4B where the normalized ECM volume is depicted changing with time for various values of *k*_2_. The dashed curves represent the corresponding normalized cellular volumes.

Note that with the higher *k*_2_ the ECM regenerates closer to pre-wound value, while in the lowest case of *k*_2_ the ECM ends up overproduced. If we interpret the additional ECM volume as scarring, then the faster movement (higher *k*_2_) of the ECM leads to the results closer to the original tissue structure.

### Tissue regeneration model in the presence of vascular network

In this section we are adding a capillary network to the tissue sample in order to simulate oxygen distribution in the sample. This means including the diffusion equation for oxygen, i.e. Eq (20), to the system of equations, subject to boundary conditions (21) at the capillary locations. As opposed to the previous examples, the oxygen partial pressure is now going to explicitly affect the cell proliferation.

**Fig 5:**
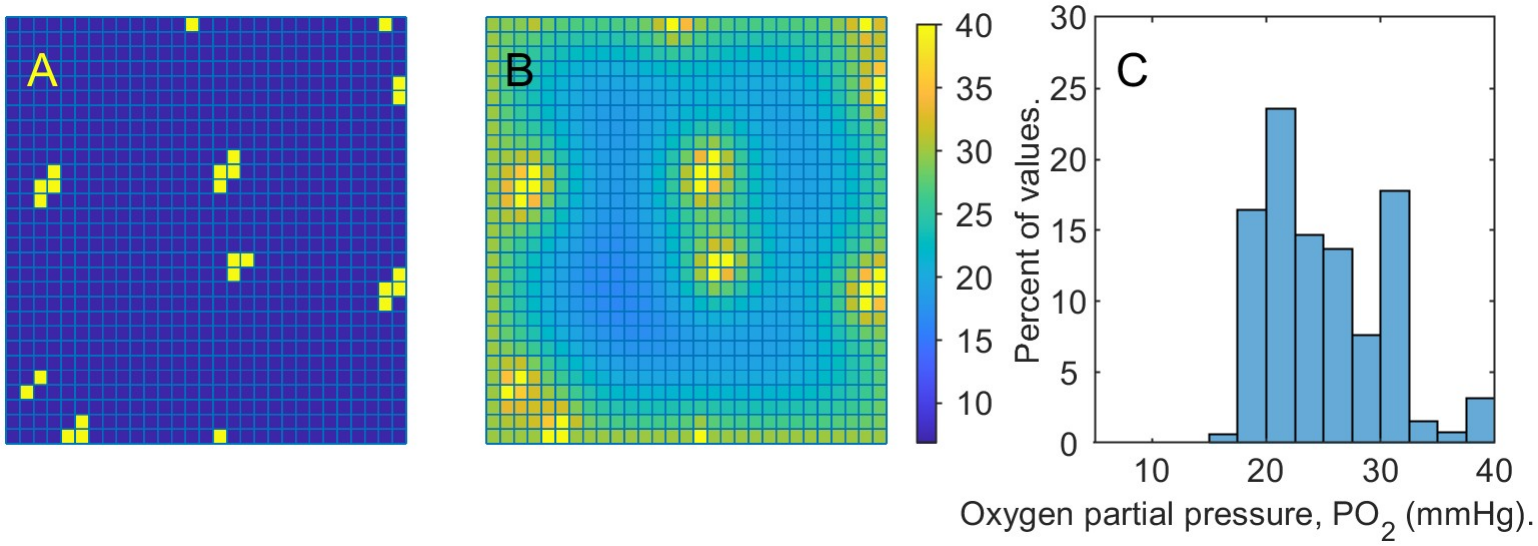
Oxygenated tissue. A: yellow squares mark the presence of capillary sources. B: oxygenation levels of the domain with the capillary sources as in the picture on the left and no damaged cells. Lower values of *PO*_2_ correspond to darker blue. As *PO*_2_ increases, the color in the picture turns yellow. The most bright yellow squares can be identified with capillary positions. C: *PO*_2_ histogram corresponding to the tissue sample for the oxygenation map depicted in the center.

The vascular system consisting of a few capillary sources is depicted in Fig 5A. Fig 5B shows the corresponding distribution of the oxygen partial pressure in the domain. The histogram in Fig 5C demonstrates that the tissue sample is well oxygenated, i.e. the *PO*_2_ value are well above the hypoxic threshold of 10 *mmHg*.

Eq (20) is solved for *o* (here standing for the oxygen partial pressure *PO*_2_), subject to the boundary *PO*_2_ value $\bar{o}=30\;mmHg$ and capillary *PO*_2_ value $\tilde{o}=40\;mmHg$, with the diffusion coefficient $\hat{D}=173$ and a parameter *δ* = 0.5. The production function is set to zero (the source of oxygen is the capillary network) and the consumption function is defined as *ou*_0_ *c*_1_ *u*_1_. If we assume the constant base consumption rate of *PO*_2_ by cells and additional consumption due to cell division and growth, we can define

$$c_{1}=\ell_{1}+\ell_{2}b_{1},$$

where *b*_1_ was introduced earlier as the cell growth rate and *ℓ*_1_, *ℓ*_2_ are positive constants.

In this example we introduce a population of “dead” cells in addition to the components introduced earlier. The reasons for including such a population are: 1) we will allow live cells to die if tissue is hypoxic, 2) it takes time for dead cells to disintegrate and become part of the interstitial fluid.

In summary, let *u*_0_ represent the volume fraction of the interstitial fluid, *u*_1_—live cells, *u*_2_—dead cells, *u*_3_—the ECM. Define *g*_0_ = −*g*_1_ − *g*_2_ − *g*_3_, where *g*_1_ = *b*_1_ *u*_1_ − *d*_1_ *u*_1_ is the net rate of change of population *u*_1_, live cells, *g*_2_ = *d*_1_ *u*_1_−*d*_2_ *u*_2_ is the net rate of change of population *u*_2_, dead cells, and *g*_3_ = *b*_3_ is the net rate of change of the ECM volume fraction.

For the cells to reflect the dependence of their well-being on *PO*_2_, we define a decay function dependent on *PO*_2_ by

$$d_{1}=\hat{d_{1}}\frac{{\left(u_{0}^{opt}-u_{0}\right)}_{+}}{u_{0}^{opt}}+\frac{{\tilde{d}}_{1}}{2}(1-\text{tanh}(R_{1}\left(o-T_{1}\right)))$$

and a growth function by

$$b_{1}=\frac{b_{1,\text{max}}}{2}u_{0}{\left\|\nabla \left[{\left(u_{0}-u_{0}^{opt}\right)}_{+}\right]\right\|}_{2}(1+\text{tanh}(R_{2}\left(o-T_{2}\right))).$$

**Fig 6:**
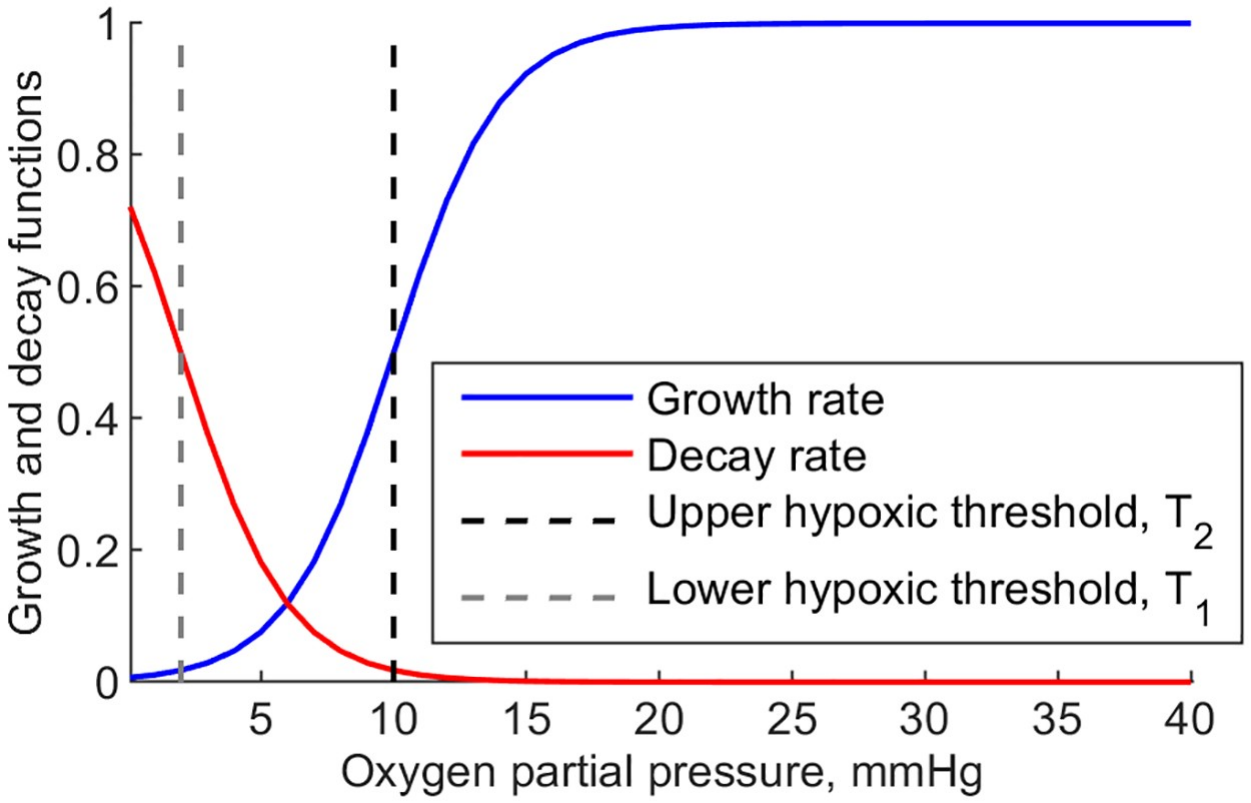
Growth and decay as functions of oxygen partial pressure.

Fig 6 shows the curves representing dependence of the growth and decay functions on oxygen partial pressure. For simplicity we set $b_{1,\text{max}}u_{0}{\left\|\nabla \left[{\left(u_{0}-u_{0}^{opt}\right)}_{+}\right]\right\|}_{2}=1$, ${\hat{d}}_{1}=0$ and ${\tilde{d}}_{1}=1$. The growth function (blue) increases towards its maximum with the increase in oxygen partial pressure. The steepness of the increase is controlled by a numerical parameter *R*_2_, and the inflection point is located at the upper hypoxic threshold parameter *T*_2_ = 10 *mmHg*. The decay function (red) decreases with oxygen partial pressure. Its steepness is controlled by a numerical parameter *R*_1_, and the inflection point is located at the lower hypoxic threshold *T*_1_ = 2 *mmHg*.

In addition to the explicit oxygen dependent term, the decay function reflects that nutrients are supplied to cells by the interstitial fluid, and its absence increases the decay rate.

The ECM growth rate is defined as in previous examples

$$b_{3}=b_{3,\text{max}}u_{0}u_{1}{\left(u_{3}^{opt}-u_{3}\right)}_{+},$$

and we will continue to assume that there is no additional damage to the ECM during the simulation, i.e, *d*_3_ = 0. The rate of dead cell disintegration *d*_2_ = 0.5 is constant everywhere in the domain and in all of the experiments in this example. We summarise the parameters involved the experiments of this subsection in Table 2.

**Table 2:** Default parameter values for the experiments in tissue regeneration model in the presence of vascular network.

Parameter	Notation	Value	Units
Live Cell Growth Rate Coefficient	*b*_1,max_	0.04	*day* ^−1^
ECM Growth Rate Coefficient	*b*_3,max_	2	*day* ^−1^
Live cell decay rate coefficients	${\hat{d}}_{1}$	0.01	*day* ^−1^
	${\tilde{d}}_{1}$	0.01	*day* ^−1^
Dead cell decay rate	*d*_2_	0.5	*day* ^−1^
ECM decay rate	*d*_3_	0	*day* ^−1^
Live cell strain-stress coefficient	*k*_1_	500	*kg* *mm*^2^/*day*^2^
Dead cell strain-stress coefficient	*k*_2_	600	*kg* *mm*^2^/*day*^2^
ECM strain-stress coefficient	*k*_3_	400	*kg* *mm*^2^/*day*^2^
Friction coefficients	${\hat{\alpha }}_{ij}$	0.8 × 10^6^	*kg*/*day*
Optimal Interstitial Fluid	$u_{0}^{opt}$	0.25	–
Volume Fraction			–
Optimal ECM Volume Fraction	$u_{2}^{opt}$	0.05	–
Domain grid size	*h*	0.01	*mm*
Time step size	Δ*t*	0.25	*day*
Diffusion coefficient	$\hat{D}$	173	*mm*^2^/*day*
Diffusion parameter	*δ*	0.5	–
*PO*_2_ boundary value	$\bar{o}$	30	*mmHg*
*PO*_2_ capillary value	$\tilde{o}$	40	*mmHg*
Base consumption rate	*ℓ*_1_	4 × 10^4^	*day* ^−1^
Additional consumption rate	*ℓ*_2_	2 × 10^4^	–
Lower hypoxic threshold	*T*_1_	2	*mmHg*
Upper hypoxic threshold	*T*_2_	10	*mmHg*
Numerical Parameters	*R*_1_	0.25	–
	*R*_2_	0.25	–

Just as in Examples 1.a-1.d, we set up the initial conditions for *u*_1_ to be 0.7 everywhere in the domain except for the set *B*, the wound. Additionally, we “fill the wound” with dead cells now, i.e., we set the volume fraction of dead cells at the the locations in the set *B* to 0.7. We run the simulation under the *PO*_2_ conditions displayed in Fig 5.

**Fig 7:**
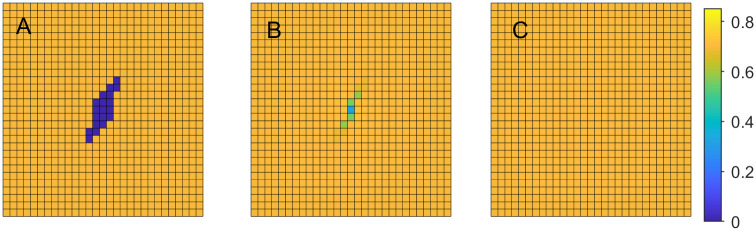
Tissue regeneration under normoxic conditions. Cellular volume fraction *u*_1_ at time steps *t* = 1, 11, 21. A: time step *t* = 1, B: time step *t* = 11, C: time step *t* = 21.

Fig 7 illustrates the healing process providing snapshots of live cell volume fractions over the domain at time steps 1, 26 and 51. The oxygen partial pressure, *PO*_2_, depicted in Fig 5, remains similar to the initial distribution throughout the simulation.

**Fig 8:**
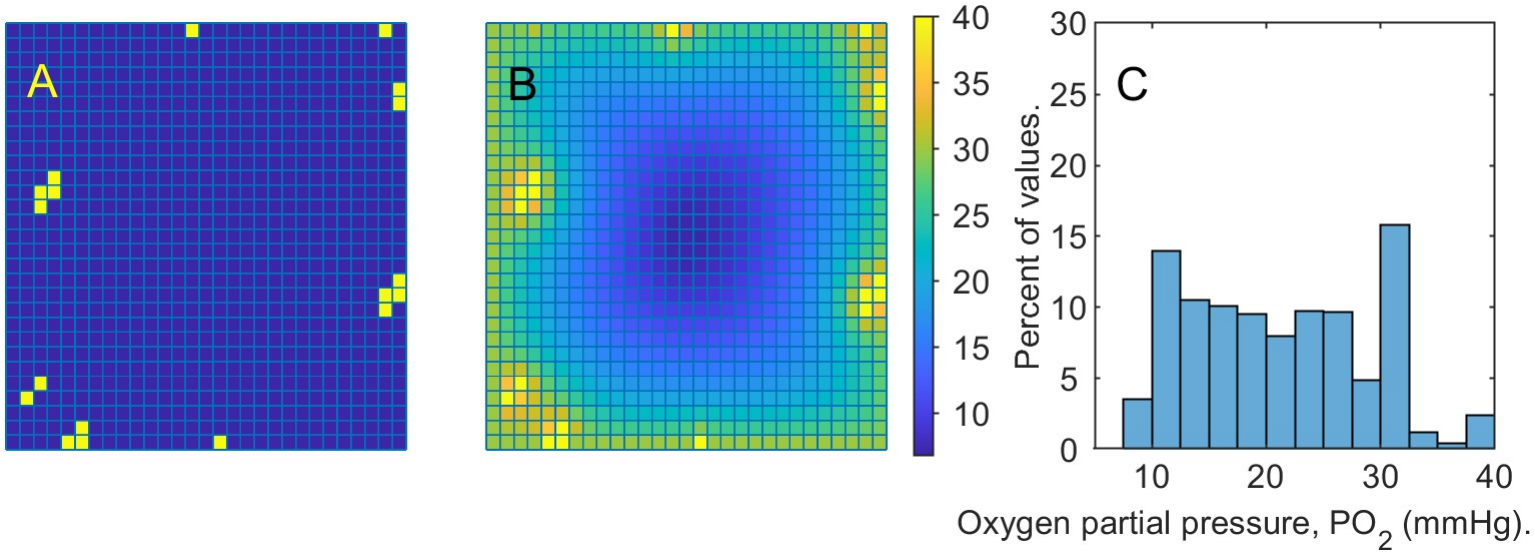
Oxygenated tissue with hypoxic regions. Left: yellow squares mark the presence of capillary sources. Center: oxygenation levels of the domain with the capillary sources as in the picture on the left and no damaged cells. Darkest blue indicates hypoxic locations. Right: *PO*_2_ histogram corresponding to the tissue sample in the center. Values below 10 are considered hypoxic.

**Fig 9:**
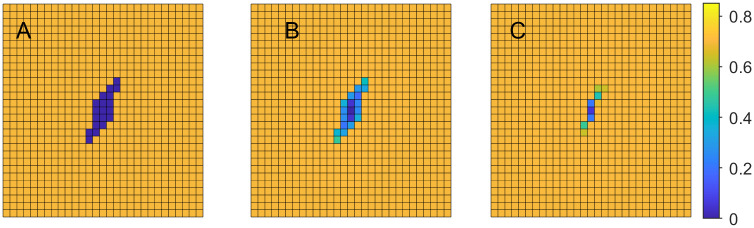
Tissue regeneration under hypoxic conditions. Cellular volume fraction *u*_1_ at time steps *t* = 1, 11, 21. A: time step *t* = 1, B: time step *t* = 11, C: time step *t* = 21. These results demonstrate that the healing process in hypoxic tissue is much slower, compared to well oxygenated tissue.

**Fig 10:**
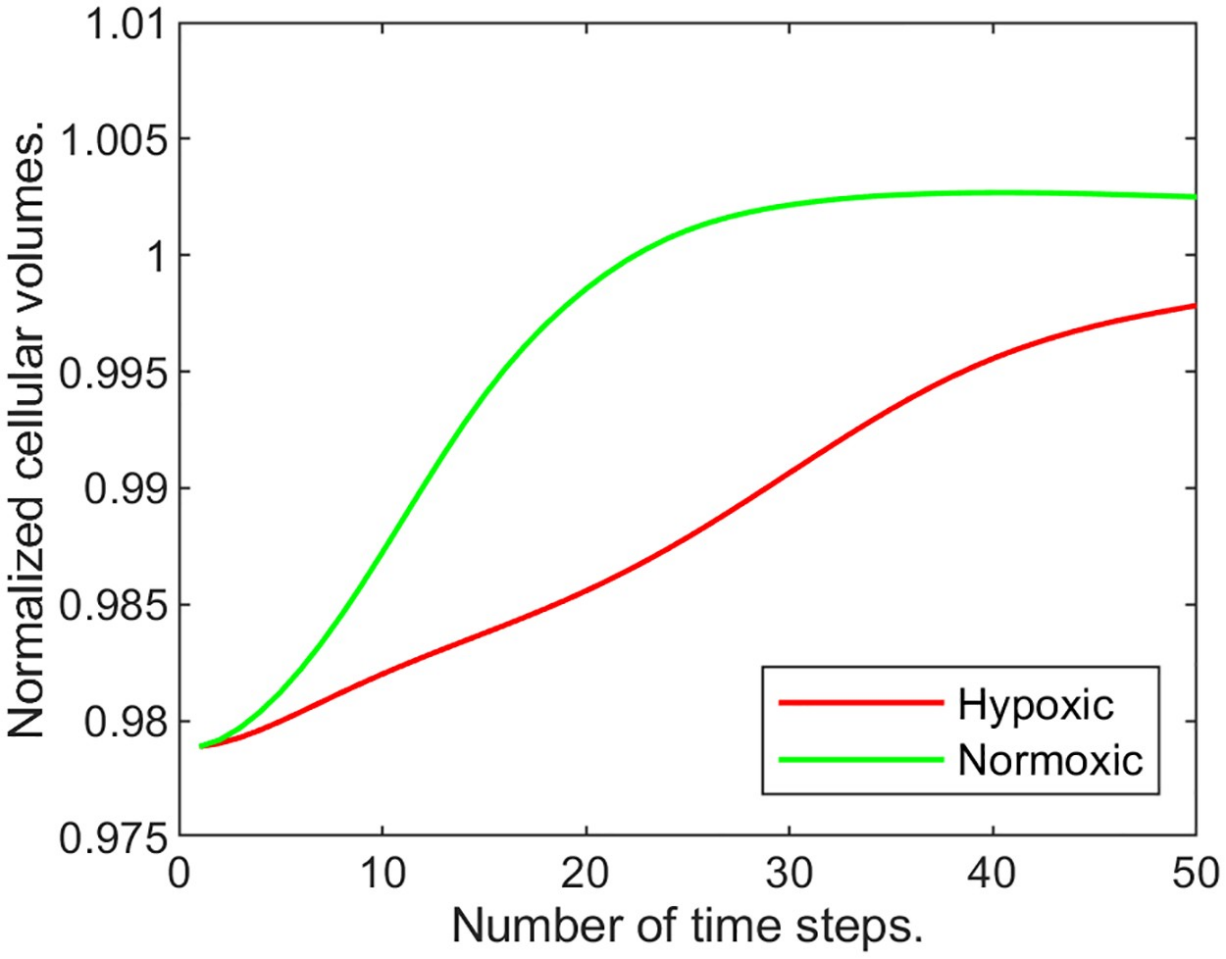
Normalized cellular volumes of live cells changing with time. Green line: results of the simulation under normoxic conditions. Red line: results of the simulation under hypoxic conditions.

Next, we remove the capillaries closest to the damaged region. Fig 8 presents the remaining capillaries, the resulting oxygenation map, and the corresponding *PO*_2_ distribution. The histogram reveals the presence of a hypoxic region, *PO*_2_ < 10 *mmHg*. We repeat the experiment and depict the changes in volume fraction of live cells at time steps 1, 26, 51 in Fig 9. The oxygen distribution remains similar to the initial setup throughout the simulation and comparing Fig 9 to the similar spread in Fig 7 we can see that under relatively unfavorable *PO*_2_ conditions tissue regeneration slows down considerably. To illustrate this further, Fig 10 compares the normalized cellular volumes of live cells in the two simulations, with and without the central capillary sources.

These examples illustrate the ability of the model to incorporate micro-environmental factors in general, and cell populations dependencies on the spacial distribution of these factors in particular.

### ECM deformation in tumorigenesis

Interactions of tumor cells with the extracellular matrix play an important role in tumorigenesis. Often tumors, especially benign ones, are surrounded by a dense capsule of fibrous tissue [35]. “Active” response of the immune system leading to the ECM reconstruction at the locations of tumor cells and “passive” response of the ECM deformations leading to local increases in the ECM density were modeled in [23, 24] for the purpose of explaining the phenomenon of tumor encapsulation.

The model in [23] was developed under the assumption of central symmetry, and as a result, the components’ volume fractions were treated as functions of time and one spatial variable. It was assumed that the tumor cells were dividing and concurrently producing the ECM. Tissue viscosity was taken into account, but vasculature, however, was not explicitly present. The tumor cell proliferation rate was proportional to the amount of the interstitial fluid, which was assumed to carry enough nutrients to sustain the growth.

The model in [24] had many similar characteristics. The main difference was that the “active” response was included in the dynamics of tumor interactions with the ECM. Some of the conclusions that followed from the simulation runs were the following: “active” response, that is, the ECM reconstruction in tumor’s presence, significantly suppresses the tumor’s growth, but without “passive” response, no capsule is formed, with high accumulation of the ECM inside the tumor.

*Here, we are using our framework* to illustrate modeling of tumor encapsulation related processes. We aim for the model to capture the growing population of tumor cells and the ECM response that this growth can illicit (both ‘passive” and “active”). The goal is to elucidate the conditions that can lead to tumor encapsulation.

Our model expands the setup in [24] to a two-dimensional case with more flexible general assumptions on the interactions between the components, addition of live cells population, and different constitutive relations for stresses. As seen in the numerical experiments presented here, our framework successfully reproduces many aspects of tumor encapsulation in a manner similar to the results in [24], as well as provides additional insights into this and related phenomena.

In the series of experiments in this section we introduce *u*_0_—volume fraction of the interstitial fluid, *u*_1_—volume fraction of live healthy cells, *u*_2_—volume fraction of live tumor cells and *u*_3_—volume fraction of the ECM. The net growth functions *g*_*i*_, *i* = 1, 2, 3 are defined in terms of proliferation and decay rates as

$$g_{1}=-{\hat{d}}_{1}u_{1}{\left(u_{0}^{opt}-u_{0}\right)}_{+}$$

$$g_{2}=({\hat{b}}_{2}u_{0}-{\hat{d}}_{2}{\left(u_{0}^{opt}-u_{0}\right)}_{+})u_{2}$$

$$g_{3}={\hat{b}}_{3}u_{0}u_{1}{\left(u_{3}^{opt}-u_{3}\right)}_{+},$$

where ${\hat{b}}_{i},\;{\hat{d}}_{i}$ are positive constants. These functions are defined under the assumptions that 1) live healthy cells may die due to unavailability of nutrients (low interstitial fluid volume fraction), and they do not proliferate; 2) cancer cells divide and grow in proportion to availability of interstitial fluid, and die otherwise; 3) the ECM growth is triggered when the ECM values fall below its normal value, is “excreted” by healthy cells, and its rate of increase depends on the availability of interstitial fluid.

As in the previous examples, the functions Ψ_*i*_, *i* = 1, 2, 3 used in the stress-strain constitutive relations, are defined by $k_{i}{\left(u_{0}^{opt}-u_{0}\right)}_{+}$, and, for simplicity, the drag coefficients ${\hat{\alpha }}_{ij}$ are assumed to be all equal.

Table 3 contains parameter values that are used in all of the following experiments, unless specified otherwise.

**Table 3:** Default parameter values and parameter ranges for the experiments in ECM deformation in tumorigenesis.

Parameter	Notation	Value	Units
Normal Cell Growth Rate	${\hat{b}}_{1}$	0	*day* ^−1^
Tumor Cell Growth Rate	${\hat{b}}_{2}$	0.9	*day* ^−1^
ECM Growth Rate Coefficient	${\hat{b}}_{3}$	50	*day* ^−1^
Normal cell decay rate	${\hat{d}}_{1}$	0.06	*day* ^−1^
Tumor cell decay rate	${\hat{d}}_{2}$	0.06	*day* ^−1^
Normal cell strain-stress coefficient	*k*_1_	1	*kg* *mm*^2^/*day*^2^
Tumor cell strain-stress coefficient	*k*_2_	1	*kg* *mm*^2^/*day*^2^
ECM strain-stress coefficient	*k*_3_	25	*kg* *mm*^2^/*day*^2^
Drag coefficients	${\hat{\alpha }}_{ij}$	10^6^	*kg*/*day*
Optimal Interstitial Fluid$u_{0}^{opt}$	0.25	–	
Volume Fraction			–
Optimal ECM Volume Fraction	$u_{2}^{opt}$	0.05	–
Domain grid size	*h*	0.01	*mm*
Time step size	Δ*t*	0.5	*day*
Numerical parameter	*β*	0.90 − 0.98	–

**Example 3.a**. In this example we ignore the ECM reconstruction, by setting ${\hat{b}}_{3}=0$, simulating just “passive” response, and vary the stress-strain coefficient *k*_3_ (*k*_3_ = 1, 5, 25). The increase of *k*_3_ impacts the excess pressure Ψ_3_ in the ECM, thus varying the intensity of the “passive” response.

We start with tumor cells located at the center of our domain (a square of side length 0.41), having the volume fraction of 0.7. Normal cell volume fraction is set to 0.7 in the rest of the domain, and the ECM volume fraction is set to 0.05 everywhere. We run simulations for 500 steps.

**Fig 11:**
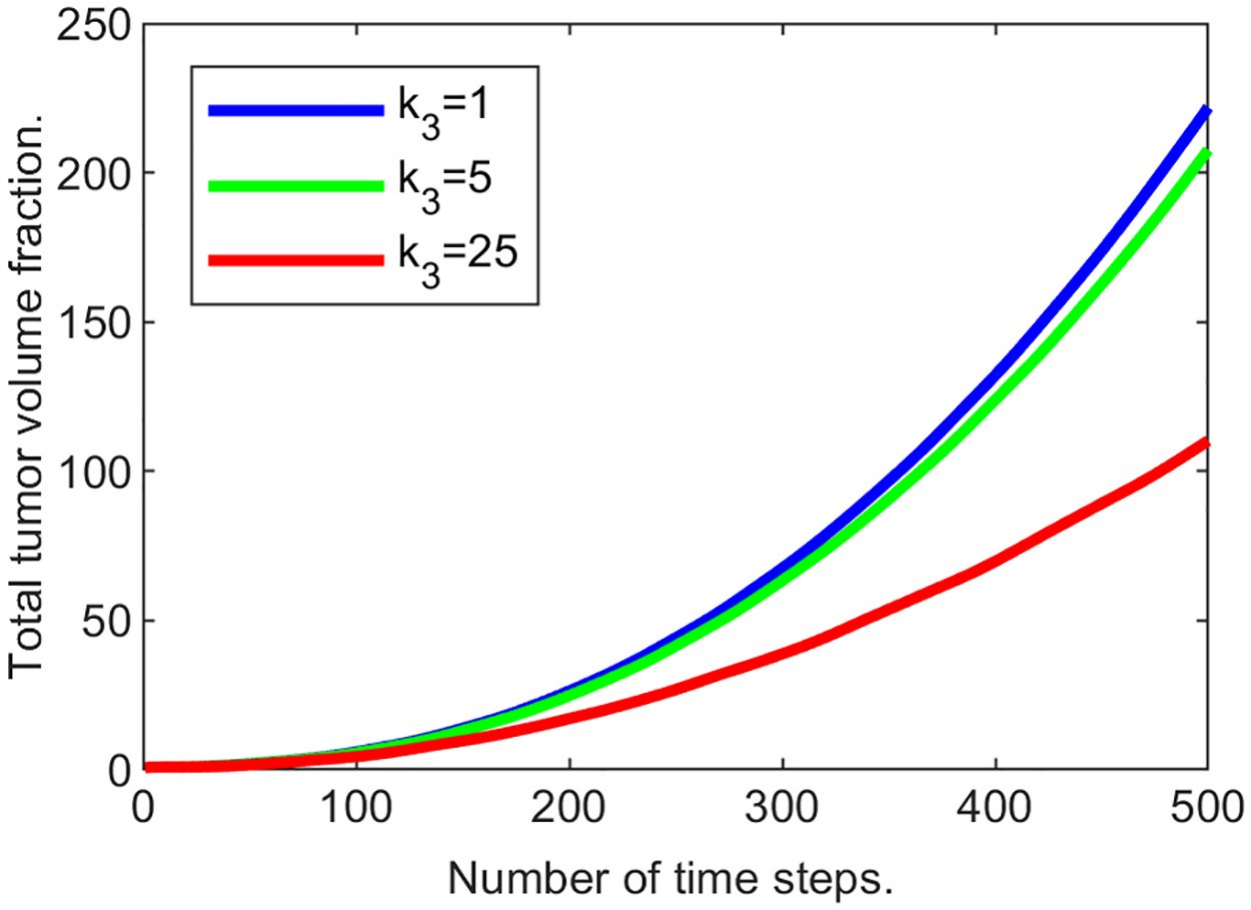
Tumor mass changing with time for various ECM strain-stress coefficients.

As seen in Fig 11 the total tumor volume (∑_*i*_ *u*_2_(*p*_*i*_) where *p*_*i*_’s are the grid locations in the domain) increases with time, and the rate of its growth decreases as *k*_3_ increases, demonstrating that “passive” response slows down the tumor expansion.

**Fig 12:**
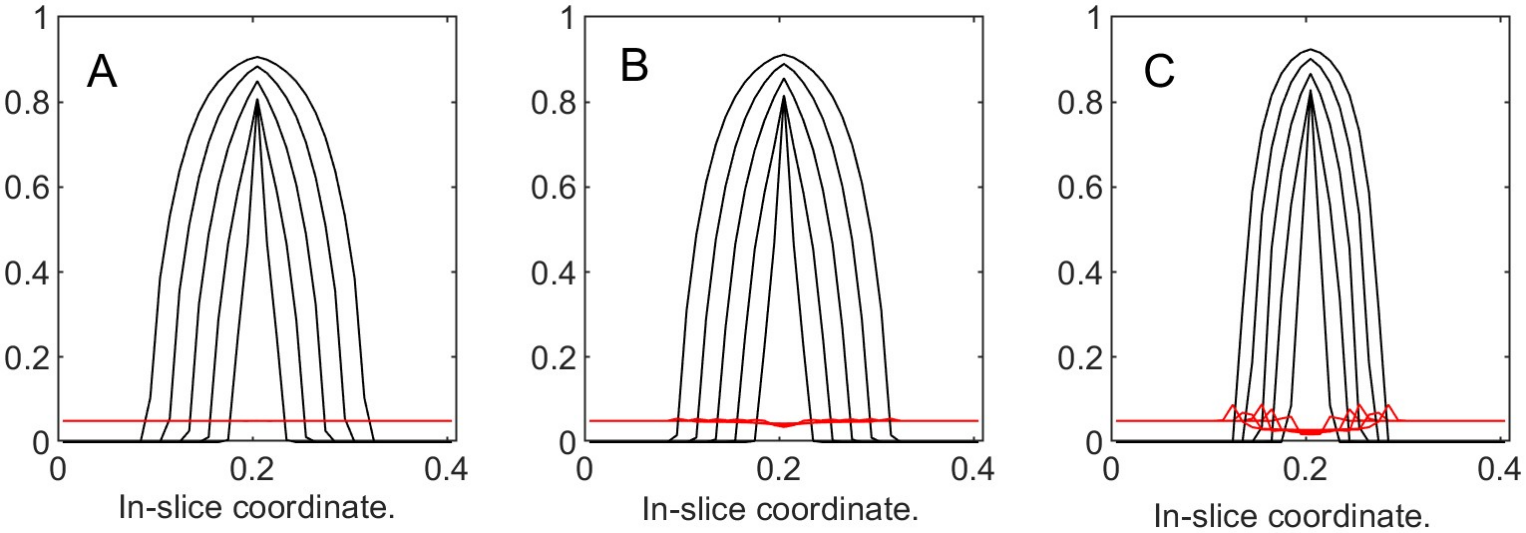
Tumor and ECM dynamics for various ECM strain-stress coefficients. Superimposed “slices” through the center of the tumor at times 100, 200,…, 500. Tumor volume fractions—black, ECM—red. A: *k*_3_ = 1 (low excess pressure). B: *k*_3_ = 5. C: *k*_3_ = 25 (high excess pressure). Note that higher *k*_3_ means higher resistance from ECM and slower tumor growth. Note also that ECM becomes visibly deformed at higher *k*_3_ values, indicating formation of a “capsule.”

**Fig 13:**
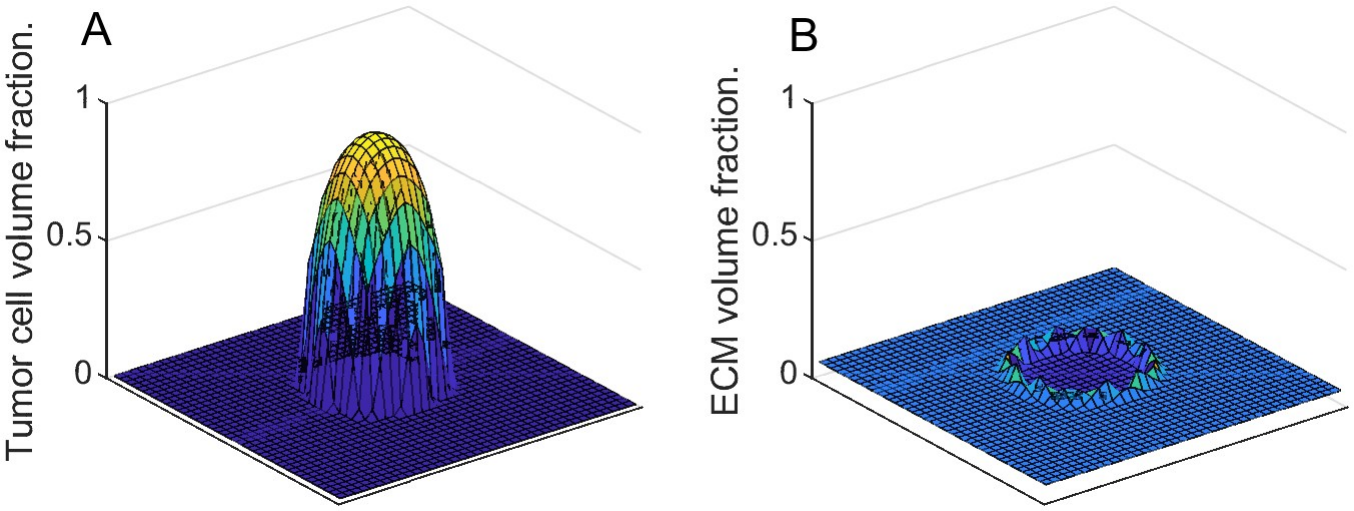
Tumor and ECM volume fractions over the domain at the last time step of the simulation with *k*_3_ = 25. A: Tumor volume fraction. B: ECM volume fraction.

To illustrate the dynamics of both tumor cells and the ECM, in Fig 12 we superimpose the “slices” of their corresponding volume fractions taken at different time steps. These slices are produced by “cutting” through the center of the domain of the corresponding functions similar to the ones in Fig 13. This representation is reasonable, since in this series of experiments we are expecting radially symmetrical solutions due to the homogeneous setup. Fig 12 demonstrates that with the increase in *k*_3_, the ECM deformations (red) become more noticeable, and the tumor’s expansion (black) is not as wide. Additionally, with larger deformations of the ECM, its levels decrease in the region around the tumor’s center, which is to be expected in the absence of the ECM reconstruction.

**Example 3.b**. In this example, in addition to the “passive” response, the “active” response is also turned on by introducing a non-zero value of the ECM growth coefficient ${\hat{b}}_{3}\in \left\{20,100,500\right\}$. Initial conditions, the length of the simulation run, and the time step are the same as before, and *k*_3_ is 25.

**Fig 14:**
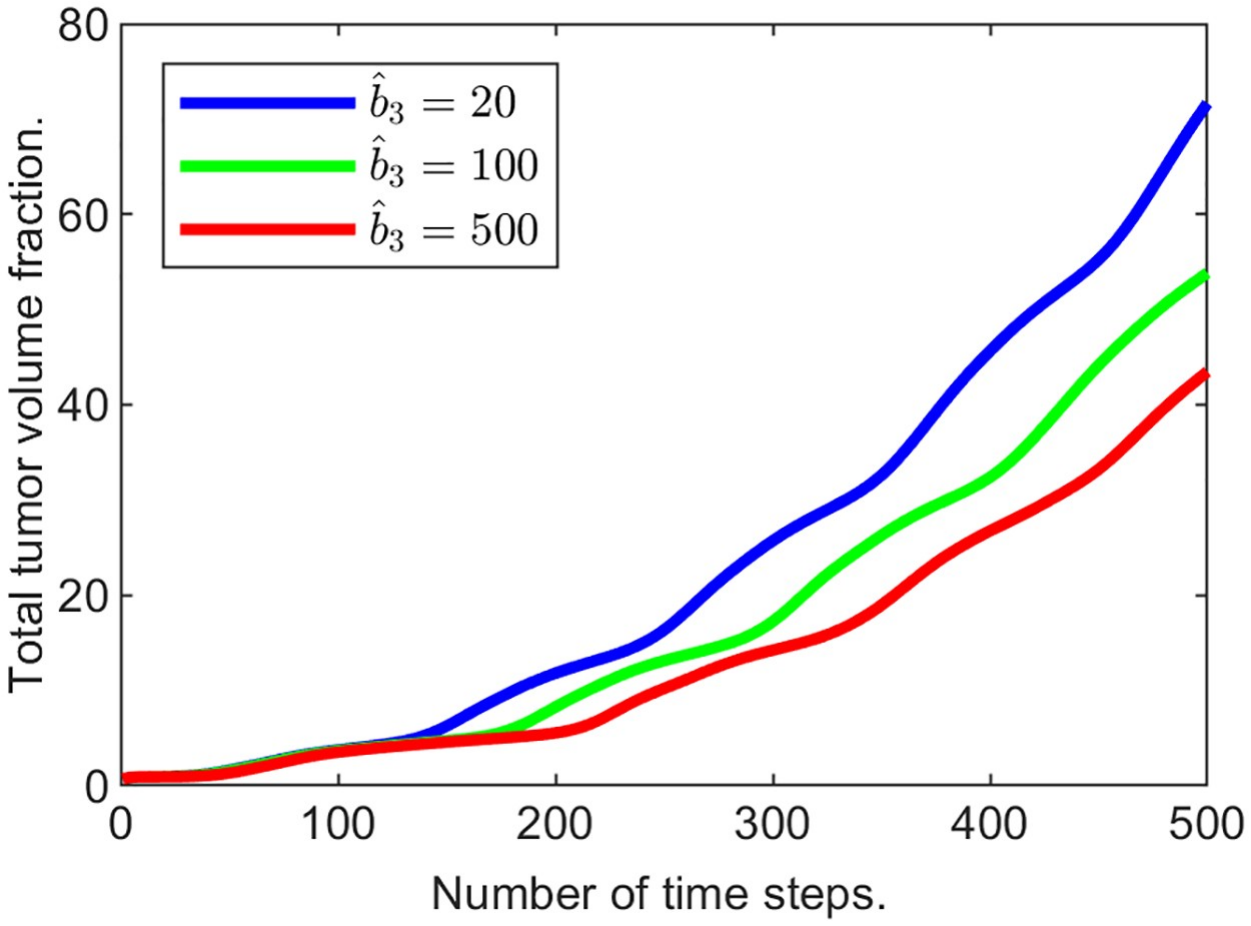
Tumor mass changing with time for various ECM remodeling coefficient values.

**Fig 15:**
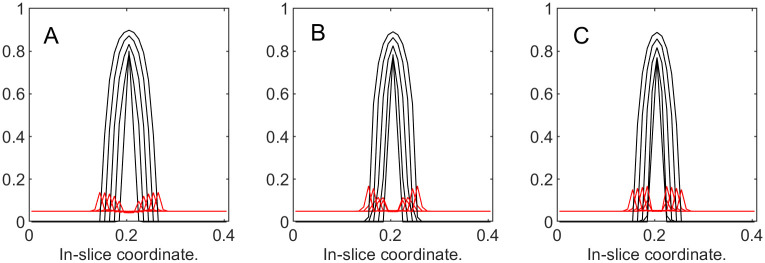
Tumor and ECM dynamics for various ECM growth coefficients. “Slices” at times 100, 200,…, 500. A: ${\hat{b}}_{3}=20$. B: ${\hat{b}}_{3}=100$. C: ${\hat{b}}_{3}=500$. Note that higher *b*_3_ values increase “active” ECM response, slowing down tumor growth.

The results are presented in Figs 14 and 15. The plot in Fig 14 demonstrates that the total tumor volume increases with time, and the rate of growth decreases as ${\hat{b}}_{3}$ increases, demonstrating that “active” response also slows down the tumor expansion. Additionally, if we compare it to the plot in Fig 11, the total volume fraction for the tumor in all three cases of “active” response inclusion are much lower than with just “passive” response. Fig 15 plots depict the slices for the tumor cells and the ECM for time steps from 100 to 500. Compared to the results in the previous example, the maximal values of the ECM are noticeably greater after the “active” response is introduced. More specifically, measured at the last time step, we have max_Ω_ *u*_3_(*t*_500_) = 0.1168 in Example 3.a (*k*_3_ = 25), and max_Ω_ *u*_3_(*t*_500_) = 0.1726 in Example 3.b, when ${\hat{b}}_{3}=500$.

We repeated the experiments with the “passive” response off, i.e. setting *k*_3_ = 0. We omit the corresponding figures here, since the results demonstrate that in the absence of “passive” response the changes in the rate of the ECM reconstruction (${\hat{b}}_{3}$ changes from 20 to 500) have no effect on the growing tumor. If cells can “pass through” the ECM without deforming it, then the capsule is never formed and the tumor is free to expand.

**Example 3.c**. In this example we allow ECM to regenerate beyond its normal optimal levels by adjusting *g*_3_ function as follows:

$$g_{3}={\hat{b}}_{3}u_{0}u_{1}{\left(\left(1+\alpha u_{2}\right)u_{3}^{opt}-u_{3}\right)}_{+}.$$

This formulation of *g*_3_ introduces an additional trigger for the ECM remodeling—presence of cancer cells. This idea is trying to capture the fact that growing tumors often synthesize abundant quantities of ECM molecules, which results in the ECM composition different from the normal tissue [41]. As *α* increases, the “active” response of the tissue to the growth of tumor cells is encouraging the ECM reconstruction to levels above normal. As a result the ECM gains the ability to encapsulate the tumor, stopping it completely from growth and expansion.

The formula for *g*_3_ captures several connections between components of the system: net growth of the ECM, *g*_3_, is proportional to *u*_0_, which demonstrates that ECM regeneration requires substrates that come from the interstitial fluid; it is proportional to the volume fraction of the live cells since it is being generated by them. The factor ()_+_ shows that the ECM remodeling is triggered only when its volume fraction falls below $\left(1+\alpha u_{2}\right)u_{3}^{opt}$; and that without the additional stimulant, *αu*_2_, the ECM can be restored to its normal value, but with a non-zero alpha, it can be generated beyond $u_{3}^{opt}$.

**Fig 16:**
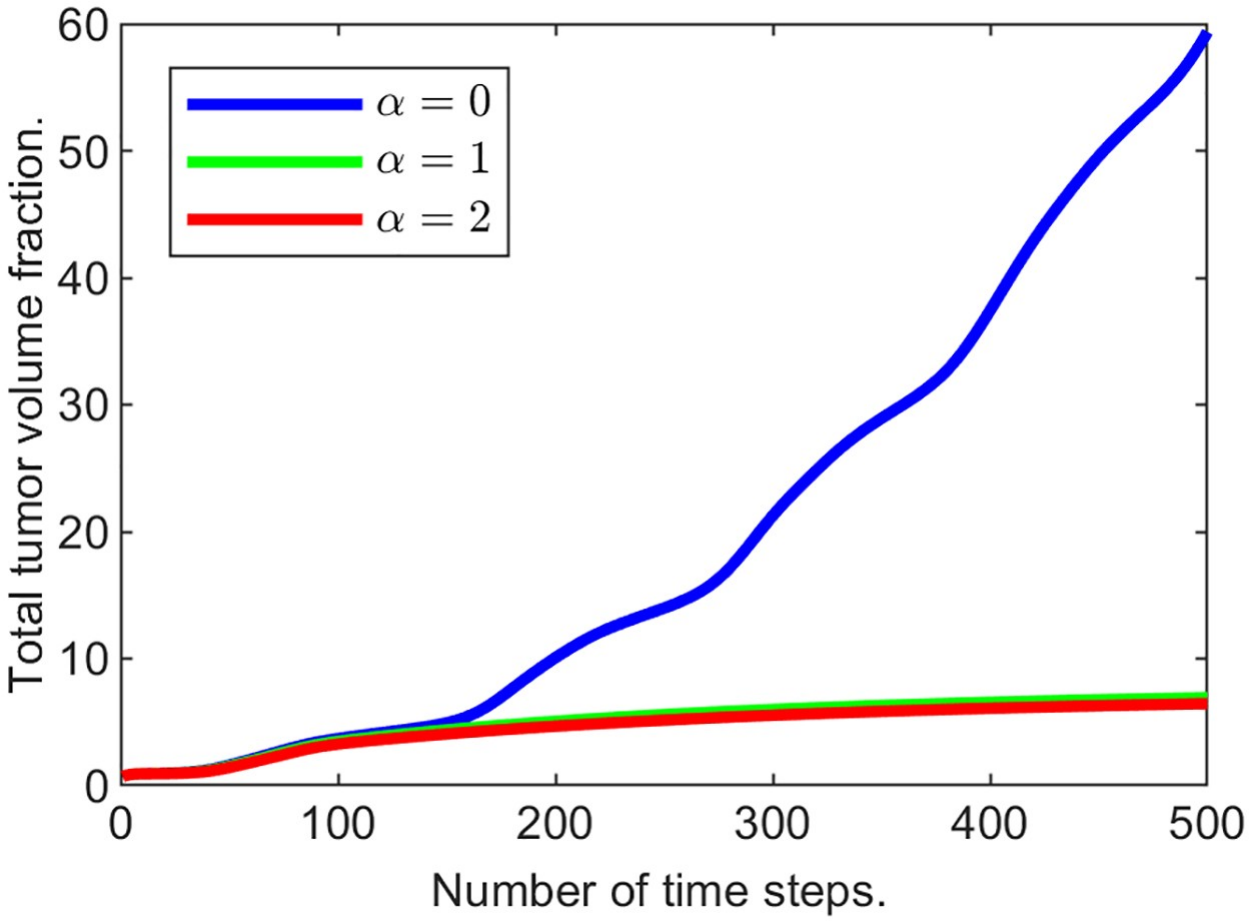
Tumor mass changing with time for various optimal ECM volume fraction values.

**Fig 17:**
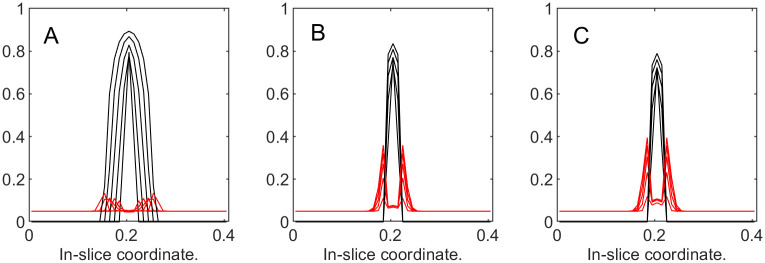
Tumor and ECM dynamics for various ECM active response coefficients. “Slices” at times 100, 200,…, 500. A: *α* = 0. B: *α* = 1. C: *α* = 2. Note that as *α* increases, tumor becomes noticeably encapsulated.

We run three experiments varying *α*. Figs 16 and 17 illustrate the results when *α* = 0, 1, 2. For each experiment we computed the maximal value of $\left(1+\alpha u_{2}\right)u_{3}^{opt}$ at the last time step. Naturally, when *α* = 0, the value of $u_{3}^{opt}$ is unchanged, i.e., 0.05. When *α* = 1, ${\text{max}}_{\Omega }\left(1+\alpha u_{2}\right)u_{3}^{opt}=0.1336$, and when *α* = 2, ${\text{max}}_{\Omega }\left(1+\alpha u_{2}\right)u_{3}^{opt}=0.1290$, showing maximal ECM volume fraction increase of 167% and 158% respectively.

**Fig 18:**
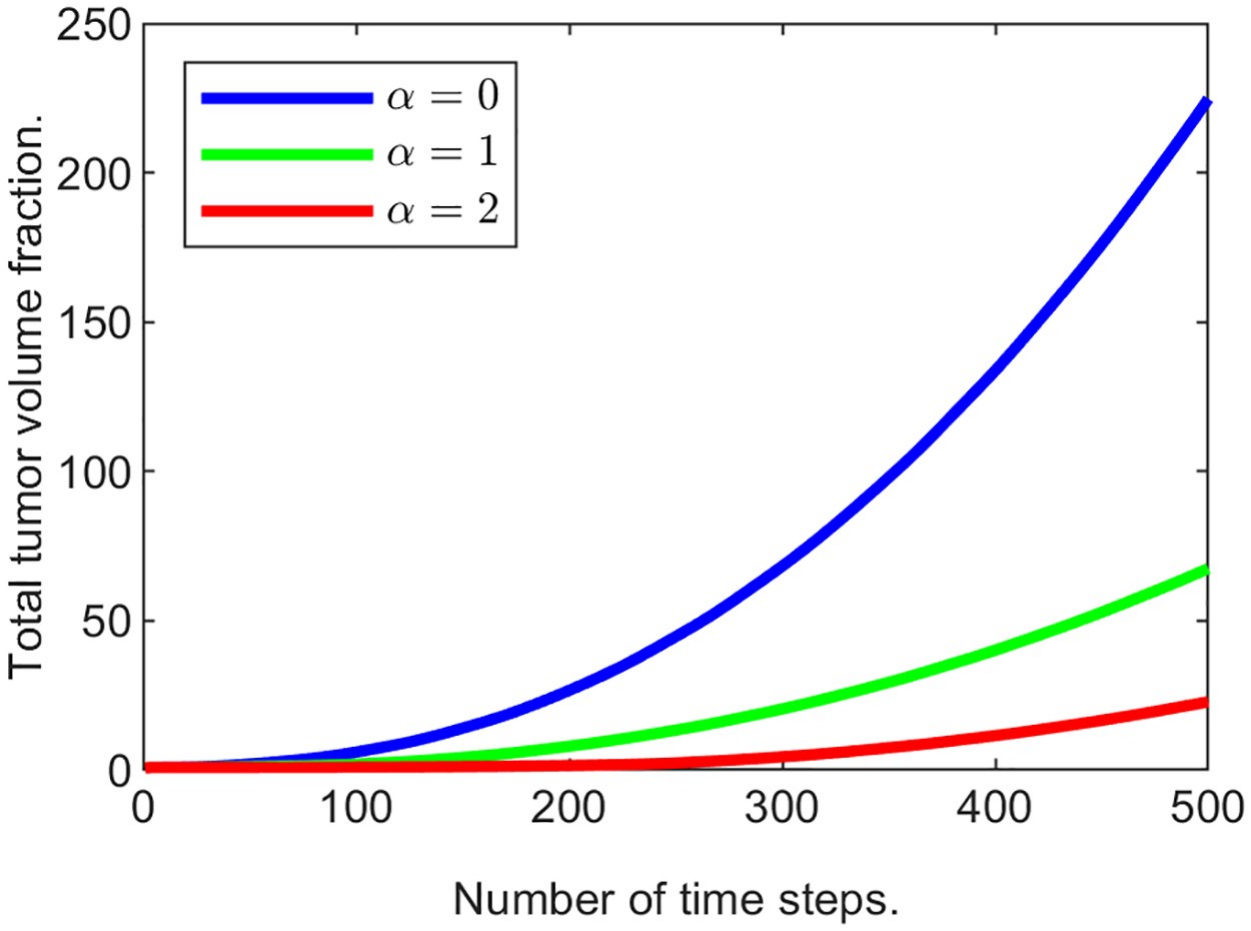
Tumor mass changing with time for various *α* with “passive” response turned off.

**Fig 19:**
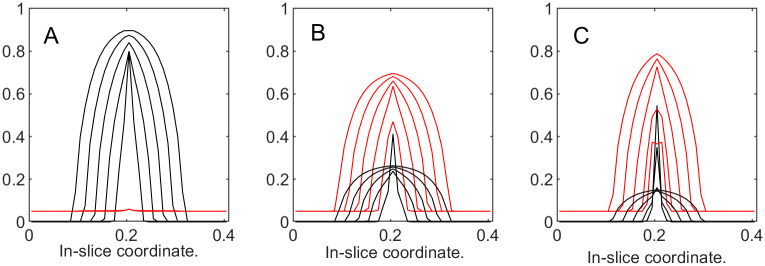
Tumor and ECM dynamics for various ECM active response coefficients. “Slices” at times 100, 200,…, 500. A: *α* = 0. B: *α* = 50. C: *α* = 100. Note that even without “passive” response, increasing $u_{3}^{opt}$ still leads to encapsulation.

To complete this example we repeated the experiment without “passive response”, at the same time taking the idea of increasing the ECM optimal value to an extreme: we run the simulations with *α* = 0 (default), *α* = 50 (4 times higher that default), and *α* = 100 (16 times higher than default). Fig 18 shows that the increase in ECM’s active response beyond normal slows down the tumor growth. Upon a closer look, Fig 19 shows that even without “passive response”, the ECM can encapsulate the tumor. Without the ability to deform, ECM simply takes over the volume initially occupied by tumor.

**Example 3.d**. In all the previous examples we set the initial ECM values to be homogeneous throughout the domain. In the example that follows we are illustrating the behavior of the system under a non-homogeneous ECM setup. The initial ECM values are defined by

$$u_{3}{|}_{t=0}=\frac{\zeta }{40}\left(\text{sin}\left(100x\right)+\text{sin}\left(100y\right)\right)+0.1+r\left(x,y\right),$$

subject to the boundary value of 0.1. Here *r*(*x*, *y*) are random numbers between −0.0012 and 0.0012. Here we use the sine functions and random values to introduce variability to the ECM volume fraction. Other formulas can be used here, we chose this particular one for ease of control over the amplitude and periodicity.

Moreover, we assume that the normal state of the ECM is also non-homogeneous and the initial conditions reflect the normal tissue architecture, i.e., $u_{i}^{opt}=u_{i}{|}_{t=0},i=0,3$. A reminder, that this, in turn, influences many aspects of the system, including components’ velocities and rates of growth and decay.

**Fig 20:**
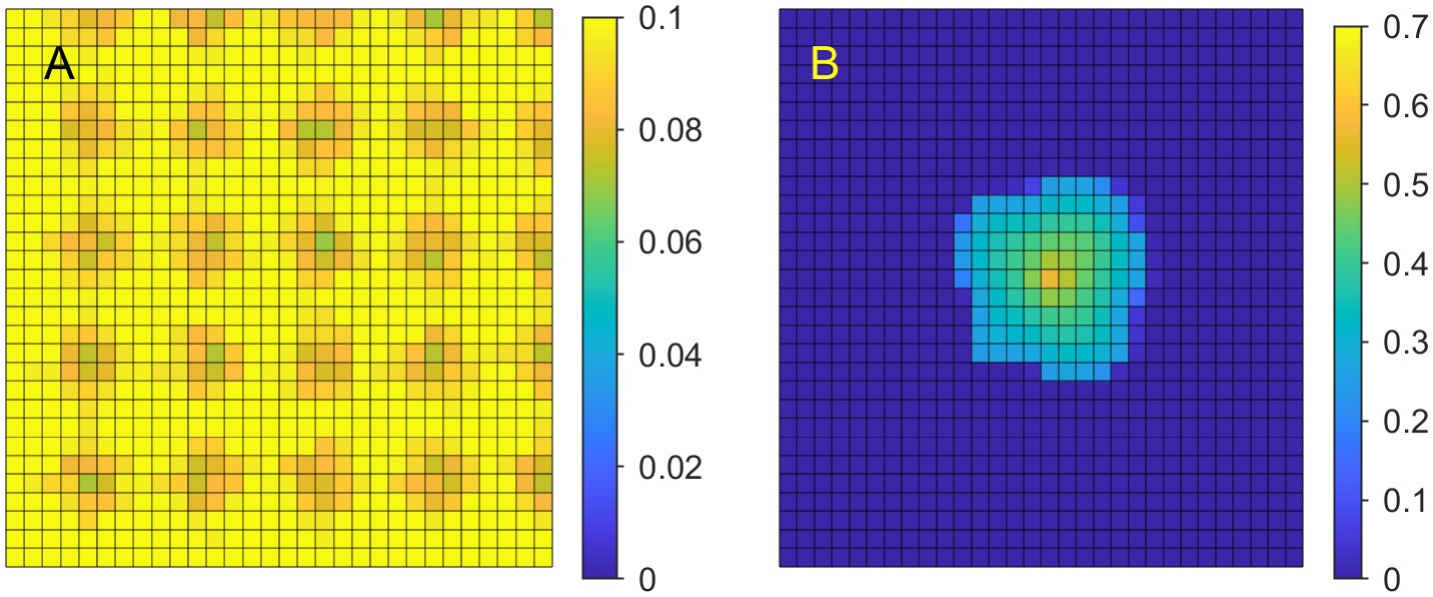
The ECM and tumor volume fractions over the domain. A: Non-homogeneous ECM. B: Asymmetrical tumor shape as a result of non-homogeneous ECM.

We run simulations with *ζ* = 0.5 (with the tumor cell proliferation rate ${\hat{b}}_{2}=1.2$) for 500 steps. The results are presented in Fig 20 and illustrate how (in the current configuration) the non-homogeneity in the ECM is influencing the shape of the tumor.

Further examples can be created to illustrate this modeling framework’s potential. Additional elements can be easily incorporated to address various aspects of tumorigenesis. For instance, one can include vasculature changes (reflecting angiogenesis and capillary damage), additional cell populations (such as immune system cells, fiberblasts, or multiple tumor subpopulations), additional chemical fields (such as ECM degrading proteins, growth factors, or drugs). We do not include these examples here for the sake of brevity.

### Model validation and accuracy

In this subsection we demonstrate model accuracy and some of its predictive power using re-scaled data from the clinical results in [6]. Below we set up two examples: 1) given time-tumor volume data we estimate tumor cell parameters, and then run a simulation with these parameters to show the accuracy of the model; 2) we estimate the parameters using partial data, and use the model with these parameters to predict the tumor volume values for the rest of the data points. The data in the two examples are generated based on the clinical data as follows. The tumor volumes are first estimated from the supplementary materials in [6]. They are then adjusted for the projection from 3D to 2D (each tumor volume is raised to the power 2/3). Finally, for the uniformity of our experiments, we scale it down for the domain and mesh sizes similar to the experiments in the subsections above. Tables 4 and 5 list the data.

**Table 4:** Tumor size data set 1. Scaled down from the clinical results in [6].

Time, in days	*t*_*i*_	0	7	11	12	13
Total volume fraction of cells in the domain	*v*_*i*_	1	13.57	46.30	47.27	58.72
Time, in days	*t*_*i*_	14	15	18	19	
Total volume fraction of cells in the domain	*v*_*i*_	68.75	77.33	109.77	122.15	

**Table 5:** Tumor size data set 2. Scaled down from the clinical results in [6].

Time, in days	*t*_*i*_	0	5	6	7	11	12
Total volume fraction of cells in the domain	*v*_*i*_	1	13.57	14.46	18.57	40.74	54.29
Time, in days	*t*_*i*_	13	14	15	18	19	
Total volume fraction of cells in the domain	*v*_*i*_	61.73	71.93	86.18	118.50	125.15	

In both examples our model’s components are the interstitial fluid, normal cells, tumor cells, and the ECM. For simplicity we assume that the normal cells do not proliferate, nor do they die. We also assume that there is no ECM degradation and no ECM reconstruction. To sum up, the set up is similar to the one in ECM Deformation and Tumorigenesis experiments with ${\hat{d}}_{1}={\hat{b}}_{1}={\hat{d}}_{3}={\hat{b}}_{3}=0$. The stress-strain coefficient for normal cells and the ECM, drag coefficients, optimal volume fractions for the ECM and the interstitial fluid are the same as listed in Table 3. The mesh size is 0.01 and the time step is 0.25. Finally, the numerical parameter *β* = 0.9. The proliferation and decay rates for tumor cells, ${\hat{b}}_{2}$ and ${\hat{d}}_{2}$, as well as tumor cells strain-stress coefficient *k*_2_ are estimated using MATLAB’s *lsqcurvefit* to minimize

$$S=\sum_{i=1}^{9}{\left(v_{i}-M\left(t_{i}\right)\right)}^{2},$$

where *M*(*t*_*i*_) is the value produced by the model at time *t*_*i*_.

The initial conditions are similar to the ones in Example 3.a. We run the simulation for 80 time steps (the last data point corresponds to 19 × 4 + 1 = 77th time step).

**Fig 21:**
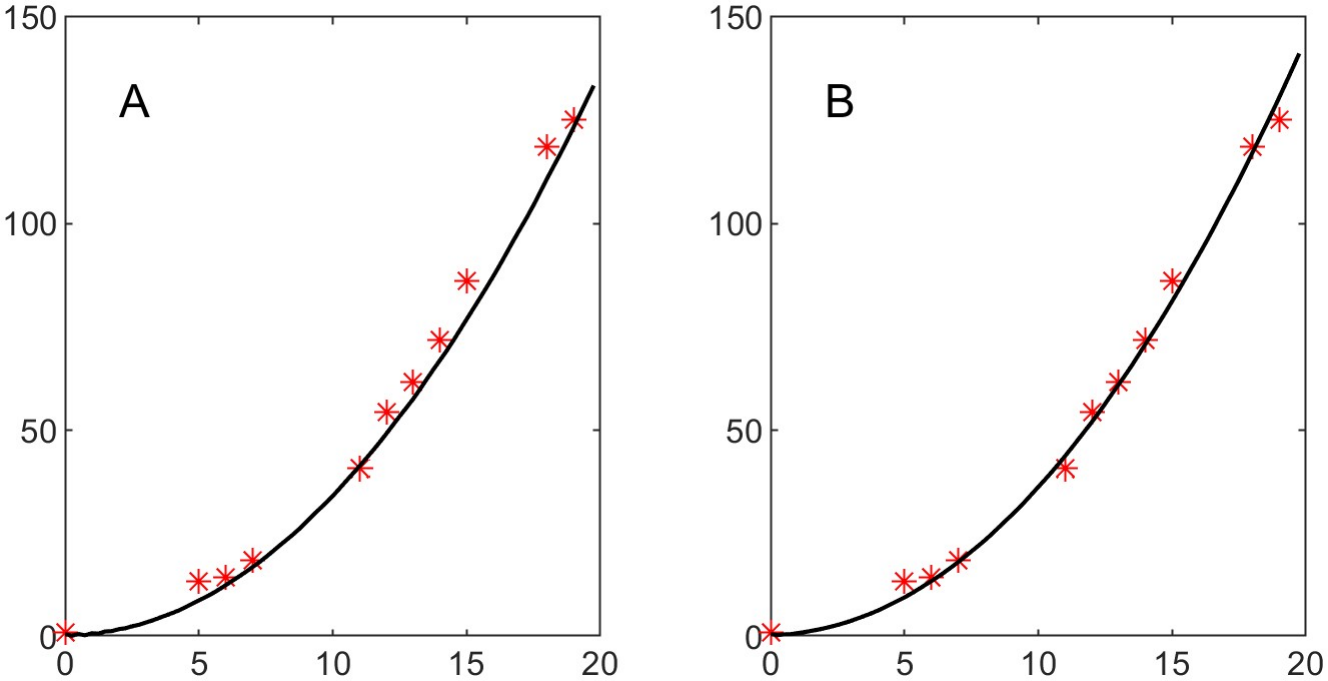
Tumor volume fraction growth over time. Black curve represents the model, red asterisks represent the data. A: Nonlinear least squares fit for data set 1. The coefficient of determination is 0.9963. B: Nonlinear Least Squares Fit for Data Set 2. The coefficient of determination is 0.9951.

**Example 4.a**. The least squares minimization procedure applied to data set 1 leads to the parameter values ${\hat{b}}_{2}=13.20$, ${\hat{d}}_{2}=0.00$, and *k*_2_ = 17.83. Running the simulation with these parameter values results in the tumor growth depicted in Fig 21A, smooth curve, where we included the data points depicted by red asterisks. The visually satisfying fit is supported by the high value of the coefficient of determination $1-\frac{\sum_{i=1}^{9}{\left(v_{i}-M\left(t_{i}\right)\right)}^{2}}{\sum_{i=1}^{9}{\left(v_{i}-\bar{v}\right)}^{2}}=0.9963$, where $\bar{v}$ is a simple mean of the data set ${\left\{v_{i}\right\}}_{i=1}^{9}$.

The least squares minimization procedure applied to data set 2 leads to the parameter values ${\hat{b}}_{2}=11.57$, ${\hat{d}}_{2}=0.83$, and *k*_2_ = 35.95. Running the simulation with these parameter values results in the tumor growth depicted in Fig 21B, black smooth curve, where we included the data points depicted by red asterisks. The visually satisfying fit is supported by the high value of the coefficient of determination 0.9951.

In [6] the authors perform similar experiments with nine classical mathematical models for description and prediction of tumor growth. The reported values for the coefficients of determination range from 0.64 to 0.98.

**Example 4.b**. In this example the least square procedure is applied to a subset of our data. The resulting parameters are used in a simulation of tumor growth and the values produced by the simulation are compared to the data not used in the parameter estimation.

Three experiments are run for each set. The first one does not use the last data point in the parameter estimation. The parameters found are used in a simulation to predict the tumor volume size on the last day of the data set. The prediction is compared to the data values using relative error

$$|\frac{v_{last}-M\left(t_{last}\right)}{v_{last}}|.$$

For the first data set this relative error is equal to 0.0205 versus 0.0399 when all of the data were used. For the second data set this relative error is equal to 0.0664 vs 0.0432 when all of the data were used.

The second experiment does not use the last two data points in the parameter estimation. The parameters found are used in a simulation to predict the tumor volume size on the last two days of the data set. The predictions are compared to the data values using the similar relative error.

For the first data set the relative errors are equal to 0.0464 (for *t*_8_) and 0.0415(for *t*_9_) versus 0.0398 and.0399 when all of the data were used. For the second data set these relative errors are equal to 0.0197 (for *t*_10_) and 0.0791 (for *t*_11_) vs 0.0109 and 0.0432 when all of the data were used.

The third experiment does not use the last three data points in the parameter estimation. The parameters found are used in a simulation to predict the tumor volume size on the last three days of the data set. The predictions are compared to the data values using the similar relative error.

For the first data set the relative errors are equal to 0.0341(for *t*_7_), 0.0724 (for *t*_8_), and 0.0806(for *t*_9_) versus 0.0584, 0.0398, and.0399 when all of the data were used. For the second data set these relative errors are equal to 0.0459(for *t*_9_), 0.0006 (for *t*_10_), and 0.0572 (for *t*_11_) versus 0.0572, 0.0109, and 0.0432 when all of the data were used.

The relative errors in all of the above experiments range between 0.06% and 8%.

## Conclusion

In this paper we presented a mathematical model that we developed with the goal of qualitatively and quantitatively capturing mechanical and chemical processes in soft tissues. The distinguishing characteristics of the model are:

- *Choice of state variables*. The equations for component velocities contain terms *u*_*i*_ **v**_*i*_ that are not well-defined at the locations where the respective volume fraction *u*_*i*_ is zero. Therefore, **v**_*i*_ is not a good choice of for a state variable—*u*_*i*_ **v**_*i*_ is. With the change of variables, the equations are well-defined everywhere on the domain, and the system possesses a unique solution under very simple conditions on the drag coefficients.
- *Conservation of momentum equations*. The equations derived from the principle of the conservation of momentum allow us to determine independent values for all state variables *u*_*i*_ **v**_*i*_. The approach adds significant flexibility to the model allowing each component of the model to move depending on the local conditions and interactions with other components. Many existing models lack this flexibility.
- *Introduction of optimal values for the interstitial fluid and the extracellular matrix*. The two parameters introduced into the model allow the components of the model to react to local changes in the tissue composition. These parameters provide a simple but effective way for the components to sense and react to deviations from normal, be it stresses due to tumor growth, or tissue damage. They effectively work as shortcuts for capturing the cumulative effects of complex biological signaling systems. Further tying the concept of deviation from normal to various biological signals allows for modeling of even more sophisticated phenomena.

We described the algorithm that produces a numerical solution to the system of partial differential equations that is the foundation of our model in S1 Appendix. We also addressed various issues related to the numerical methods used to approximate the solution to the system of PDE’s in S2–S4 Appendices.

We demonstrated the model’s potential in two groups of examples: tissue remodeling (with and without explicit oxygenation), and the ECM deformation under the pressure from the growing tumor.

Potential applications and direction include but are not limited to:

- Analysis of evolutionary dynamics of multiple tumor cell sub-populations under the micro-environmental stresses [36, 37].
- Study of the interplay between Warburg effect and metabolic requirements during cancer progression [36, 38–40].
- Study of the effects of drug/radiation/fasting therapy on tumor development [9, 41].
- More detailed study of wound healing and scarring aspects, such epidermal-dermal interactions [42], fibroblasts activation dynamics [43], and more.
- Extending the model to the 3D domains incorporating realistic, inherently three-dimensional, processes of angiogenesis and vasculature rearrangements.
- Introducing more complex strain-stress relationships into the model reflecting such properties viscosity, fiber orientation, and more.

In summary, we developed a modeling and computational framework that offers a rich toolset that we believe is flexible enough to serve as an instrument for studying a wide range of biological phenomena associated with soft tissue dynamics.

## Supporting information

S1 Appendix Numerical scheme, main algorithm. (PDF)

S2 Appendix Numerical solution of the diffusion equation for chemical species. (PDF)

S3 Appendix Numerical details, conservation of momentum and velocity fields. (PDF)

S4 Appendix Stability considerations and time step. (PDF)
