# Supplementary material for: Mathematical model and computational scheme for multi-phase modeling of cellular population and microenvironmental dynamics in soft tissue
Source: PLoS One. 2021 Nov 17;16(11):e0260108. doi: 10.1371/journal.pone.0260108 (PMC8598064; doi:10.1371/journal.pone.0260108)
Supplement: S1 Appendix — (PDF) [file pone.0260108.s001.pdf]

Supporting information

S1 Appendix. Numerical scheme, main algorithm. We begin this section by summarizing the model. The equations that describe time evolution of volume fractions are

$$\frac{\partial u_i}{\partial t} + \nabla \cdot (u_i \mathbf{v}_i) = g_i, \quad i = 1, ..., n+1,$$
$$u_0 = 1 - \sum_{i=1}^{n+1} u_i. \tag{S1-1}$$

The equations that describe velocities of volume fractions are

$$\left(\hat{\alpha}_{i0} u_i + \sum_{j=0, j\neq i}^{n+1} \hat{\alpha}_{ij} u_j \right) u_i \mathbf{v}_i + u_i \sum_{j=1, j\neq i}^{n+1} \left(\hat{\alpha}_{i0} - \hat{\alpha}_{ij} \right) u_j \mathbf{v}_j$$
$$= \nabla (u_i \Psi_i) - u_i \nabla \sum_{j=1}^{n+1} u_j \Psi_j, \quad i = 1, ..., n+1,$$
$$u_0 \mathbf{v}_0 = - \sum_{j=1}^{n+1} u_j \mathbf{v}_j, \tag{S1-2}$$

subject to the stresses defined by

$$\Psi_i = k_i (u_0^{opt} - u_0)_+, \quad i = 1, \cdots, n+1. \tag{S1-3}$$

Equations that describe chemical fields are

$$-\hat{D}_i u_0^{\delta} \Delta o_i + \nabla o_i \cdot (u_0 \mathbf{v}_0 - \hat{D}_i \nabla u_0^{\delta}) + o_i g_0 - r_i + c_i = 0, \tag{S1-4}$$

with i representing every chemical field in the model.

The chemical concentrations control birth-death rates of components' volume fractions and depend, in general, on a particular simulation. These functions influence the component net production rates.

To approximate the solutions of the equations above, we discretize space and time by the positive parameters h and Δt, respectively. Specifically, we discretize $\Omega = [a, b] \times [c, d]$ by introducing a mesh-size $h > 0$ and nodes $x_k = a + (k-1)h, \ k = 1, ..., N$ and $y_j = b + (j-1)h, \ j = 1, ..., M$.

Let p_{kj} denote locations at the centers of the squares $[x_k, x_{k+1}] \times [y_j, y_{j+1}]$, that is, $p_{kj} = (x_k + h/2, y_j + h/2), \ k = 1, ..., N-1, \ j = 1, ..., M-1$. Introducing these central locations allows us to approximate the spacial derivatives involved in our equations using a half step $h/2$ rather than the full grid-size h.

Eq (S1-1) are approximated by their discretized versions

$$\frac{\partial u_i}{\partial t}(p_{kj}) + \nabla \cdot (u_i \mathbf{v}_i)(p_{kj}) = g_i(p_{kj}), \quad i = 1, ..., n+1,$$

and the numerical solutions to the system of discretized equations at the locations p_{kj} are found by a method based on a Predictor-Corrector scheme also classified as a

2-stage Runge-Kutta method. This method, when applied to a system of first order ODE's $y'(t) = F(t, y(t))$, is formulated as

$$y^{(\ell+1)} = y^{(\ell)} + \frac{\Delta t}{2}(F(y^{\ell}) + F(\bar{y}^{(\ell+1)})), \tag{S1-5}$$

where $y^{(0)}$ is defined by initial conditions, $\Delta t > 0$ is a time step size and $y^{(\ell)}$ stands for a numerical approximation to $y(t^{(\ell)})$. Furthermore, $\bar{y}^{(\ell+1)}$ is an approximation of $y^{(\ell+1)}$ computed by Euler's method:

$$\bar{y}^{(\ell+1)} = y^{(\ell)} + \Delta t F(y^{(\ell)}).$$

The method is explicit, order two accurate and is strong stability preserving [2].

In our case

$$y(t) = (u_i(p_{kj}))_{i=1}^{n+1}(t)$$

and

$$y'(t) = (g_i(p_{kj}) - \nabla \cdot (u_i \mathbf{v}_i)(p_{kj}))_{i=1}^{n+1}(t) = F(t, y(t)).$$

Note that before we are able to advance from time step $t^{(\ell)}$ to $t^{(\ell+1)}$ we have to compute the velocities of the components at the moment of time $t^{(\ell)}$ using Eq (S1-2). Additionally, we need to know net production rates g_i, which are influenced by chemical fields, thus solving (S1-4). We, therefore, have to perform the following calculations at every time step $t^{(\ell)}$:

Algorithm 1. **Predictor-Corrector**

1. Given the values of the components in our system $u_i^{(\ell)},\ i = 0, ..., n+1$, compute the chemical fields for the moment of time $t^{(\ell)}$ at every location p_{kj}.
2. Using results from Step 1, compute birth/deaths rates of the components and determine $g_i^{(\ell)},\ i = 0, ..., n+1$ at every location p_{kj}.
3. Solve the system of force balance equations for $(u_i \mathbf{v}_i)^{(\ell)},\ i = 1, ..., n+1$ to have the approximations available at $p_{kj} \pm (h/2, 0)$, $p_{kj} \pm (0, h/2)$, $p_{kj} \pm (h/2, h/2)$ and $p_{kj} \pm (-h/2, h/2)$ for every interior location p_{kj}.
4. Use Euler's method to predict the values $u_i^{(\ell+1)},\ i = 1, ..., n+1$ at every interior p_{kj}, and set the boundary conditions for every boundary location.
5. Repeat Steps 1, 2, 3 using the results from Step 4 to get the predictions for $g_i^{(\ell+1)}$ and $(u_i \mathbf{v}_i)^{(\ell+1)},\ i = 1, ..., n+1$.
6. Use Eq (S1-5) to compute $u_i^{(\ell+1)},\ i = 1, ..., n+1$ in terms of the values $g_i^{(\ell)}, (u_i \mathbf{v}_i)^{(\ell)}$ from Steps 2 and 3, and the values $g_i^{(\ell+1)}, (u_i \mathbf{v}_i)^{(\ell+1)}$ from Step 5 at every interior location p_{kj}, and set the boundary conditions for every boundary location. Increment ℓ and go to Step 1.

Before we dive into the details of steps 1, 3, 5, let us describe how we approximate the derivatives involved in steps 4, 6. For simplicity, we are dropping the indices i and ℓ.

The numerical experiments performed in [1] show that the classical finite difference formulas may lead to approximations "preferring" x, y-directions. This phenomena can be observed when simulating early stages of growing tumors. In two dimensional models, a tumor is expected to grow as a circle, but the experiments in [1] produced diamond-like shapes. The following approach was proposed in [1], and we use it here as well:

Choose $0 \le \beta \le 1$, then

$$\frac{\partial (u\mathbf{v}^{(x)})_{kj}}{\partial x} \approx \beta \frac{(u\mathbf{v}^{(x)})_{k+1/2,j} - (u\mathbf{v}^{(x)})_{k-1/2,j}}{h} +$$
$$(1-\beta)\frac{(u\mathbf{v}^{(x)})_{k+1/2,j+1/2} - (u\mathbf{v}^{(x)})_{k-1/2,j-1/2}}{2h} +$$
$$(1-\beta)\frac{(u\mathbf{v}^{(x)})_{k+1/2,j-1/2} - (u\mathbf{v}^{(x)})_{k-1/2,j+1/2}}{2h} \quad \text{(S1-6)}$$

and

$$\frac{\partial (u\mathbf{v}^{(y)})_{kj}}{\partial y} \approx \beta \frac{(u\mathbf{v}^{(y)})_{k,j+1/2} - (u\mathbf{v}^{(y)})_{k,j-1/2}}{h} +$$
$$(1-\beta)\frac{(u\mathbf{v}^{(y)})_{k+1/2,j+1/2} - (u\mathbf{v}^{(y)})_{k-1/2,j-1/2}}{2h} +$$
$$(1-\beta)\frac{-(u\mathbf{v}^{(y)})_{k+1/2,j-1/2} + (u\mathbf{v}^{(y)})_{k-1/2,j+1/2}}{2h}, \quad \text{(S1-7)}$$

provide approximations of order two that are less biased towards x- and y- directions. As a result, when simulating tumor growth one observes the expected circular shapes.

Going over the steps above, 1 and 5 involve solving a second order partial differential equation, often nonlinear. In this case we use Newton's method for systems to find an approximation to the solution of the equation over a grid.

References

1. Baramidze GT. Computational multiphase modeling of tumor dynamics under microenvironmental stresses [dissertation]. Athens, GA: University of Georgia; 2017.

2. Hesthaven JS. Numerical methods for conservation laws: From analysis to algorithms. Society for Industrial and Applied Mathematics; 2017 Dec 21.
