# Supplementary material for: Mathematical model and computational scheme for multi-phase modeling of cellular population and microenvironmental dynamics in soft tissue
Source: PLoS One. 2021 Nov 17;16(11):e0260108. doi: 10.1371/journal.pone.0260108 (PMC8598064; doi:10.1371/journal.pone.0260108)
Supplement: S2 Appendix — (PDF) [file pone.0260108.s002.pdf]

Supporting information

S2 Appendix. Numerical solution of the diffusion equation for chemical species. For some modes it is important to simulate interactions between cells and chemical fields, which means that there is a need to accurately solve a PDE of the form

$$-\hat{D}_i u_0^\delta \Delta o_i + \nabla o_i \cdot (u_0 \mathbf{v}_0 - \hat{D}_i \nabla u_0^\delta) + o_i g_0 - r_i + c_i = 0.$$

Here we illustrate how to obtain an approximate solution to this problem using oxygen partial pressure, o, as an example:

$$-\hat{D} u_0^\delta \Delta o + \nabla o \cdot (u_0 \mathbf{v}_0 - \hat{D} \nabla u_0^\delta) + o g_0 + u_0 o \sum_{j=1}^{n+1} c_j u_j = 0.$$

Define the coefficient functions involved in this equation as follows:

$$a = -\hat{D} u_0^\delta,$$

$$\mathbf{b} = u_0 \mathbf{v}_0 - \hat{D} \nabla (u_0^\delta),$$

$$c = g_0 + u_0 \sum_{j=1}^{n+1} c_j u_j,$$

where a, b, c are functions of time and space,

$$g_0 = -\sum_{j=1}^{n+1} g_i,$$

and some of the rates c_j may depend on oxygen partial pressure. We rewrite the equation above as

$$a \Delta o + \mathbf{b} \cdot \nabla o + c o = 0, \ (x, y) \in \Omega, \tag{S2-1}$$

subject to Dirichlet boundary conditions

$$o|_{\partial \Omega} = \bar{o}$$

and capillary value condition

$$o|_{W \subset \Omega} = \tilde{o}.$$

Denoting $o(p_{ij})$ by o_{ij}, and using the well-known five point formula to approximate the Laplacian [1], we get

$$\Delta o_{ij} = \frac{1}{h^2} (o_{i+1,j} + o_{i-1,j} + o_{i,j+1} + o_{i,j-1} - 4 o_{ij}) + O(h^2),$$

that can be used away from the boundary of Ω, that is for $i = 2, ..., N-2$ and $j = 2, ..., M-2$.

Similarly we use finite difference formulas of order two for the first order derivatives to approximate ∇o at the interior grid points. That is, we have

$$\nabla o_{ij} = \frac{1}{2h} \begin{pmatrix} o_{i+1,j} - o_{i-1,j} \\ o_{i,j+1} - o_{i,j-1} \end{pmatrix} + O(h^2).$$

Then (S2-1), at a point p_{ij}, for $i = 2, ..., N-2$ and $j = 2, ..., M-2$, becomes

$$a_{ij}\Delta o_{ij} + \mathbf{b}_{ij} \cdot \nabla o_{ij} + c_{ij} o_{ij} = \frac{1}{h^2} a_{ij} \left(o_{i+1,j} + o_{i-1,j} + o_{i,j+1} + o_{i,j-1} - 4 o_{ij} \right) + \frac{1}{2h} \left(\mathbf{b}^x_{ij}(o_{i+1,j} - o_{i-1,j}) + \mathbf{b}^y_{ij}(o_{i,j+1} - o_{i,j-1}) \right) + c_{ij} o_{ij} + O(h^2) = 0.$$

Rearranging the terms in the equation above and dropping the error term $O(h^2)$ we get

$$\left(\frac{a_{ij}}{h^2} + \frac{\mathbf{b}^x_{ij}}{2h}\right) w_{i+1,j} + \left(\frac{a_{ij}}{h^2} - \frac{\mathbf{b}^x_{ij}}{2h}\right) w_{i-1,j} + \left(\frac{a_{ij}}{h^2} + \frac{\mathbf{b}^y_{ij}}{2h}\right) w_{i,j+1} + \left(\frac{a_{ij}}{h^2} - \frac{\mathbf{b}^y_{ij}}{2h}\right) w_{i,j-1} + \left(c_{ij} - 4\frac{a_{ij}}{h^2}\right) w_{ij} = 0, \tag{S2-2}$$

where w is the approximation of o. Multiplying all terms by h^2 we get

$$\left(a_{ij} + \frac{h}{2}\mathbf{b}^x_{ij}\right) w_{i+1,j} + \left(a_{ij} - \frac{h}{2}\mathbf{b}^x_{ij}\right) w_{i-1,j} + \left(a_{ij} + \frac{h}{2}\mathbf{b}^y_{ij}\right) w_{i,j+1} + \left(a_{ij} - \frac{h}{2}\mathbf{b}^y_{ij}\right) w_{i,j-1} + (h^2 c_{ij} - 4a_{ij}) w_{ij} = 0. \tag{S2-3}$$

The solution $\mathbf{w} = (w_{ij}),\ \ i = 2, ..., N-2,\ \ j = 2, ..., M-2$, (with appropriate equations for boundary and near the boundary points discussed below) of Eq (S2-3) approximates the solution o of Eq (S2-1) at locations p_{ij} with $O(h^2)$.

If and when g_0 and some of the rates c_{1j} depend on o, Eq (S2-3) are not linear, and we use Newton's method to compute the approximation. Before we describe the details, let us specify boundary and capillary conditions.

Define a set of indices of the points p_{ij} that lie near the boundary of Ω

$$\mathcal{B} := \{(1, j), (N-1, j),\ j = 1, ..., M-1,\ (i, 1), (i, M-1),\ i = 2, ..., N-2\}.$$

The boundary condition

$$o|_{\partial\Omega} = \bar{o}$$

can be approximated by

$$w_{ij} = \bar{o}, (i, j) \in \mathcal{B},$$

since, by Taylor's series,

$$f(x) - f(x+h) = O(h).$$

(The accuracy of implementation of boundary conditions may be further improved by using Taylor's series in two variables at $p_{1j}, j = 2, ..., M-2$:

$$o(p_{1j}) = \frac{2}{3}\bar{o} + \frac{1}{3} o(p_{2j}) + O(h^2),$$

similarly for the other three near boundary subsets, at the corner

$$o(p_{11}) = \frac{11}{12}\bar{o} + \frac{1}{12} o(p_{22}) + O(h^2)$$

and similarly for the other three corners.)

We assume that PO_2 is produced by capillary sources and define a set $\mathcal{W}$ of indices (i,j) such p_{ij} is a capillary location and set

$$w_{ij} = \tilde{o}(p_{ij}), (i,j) \in \mathcal{W},$$

where $\tilde{o}$ may change from capillary to capillary.

Let $\mathcal{A}$ be a set of indices $(i,j),\ i = 1,...,N-1,\ j = 1,...,M-1$, such that $(i,j) \notin \mathcal{B} \cup \mathcal{W}$. Define a linear map from the set of double indices $(i,j),\ i = 1,...,N,\ j = 1,...,M$, to the set of positive integers $I = (N-1)(j-1)+i$. This map allows us to switch to a single index notation and organize our variables as one-dimensional arrays. Define

$$\begin{aligned}
\mathbf{F}_I &= w_I - \bar{o}_1,\ \text{for } (i,j) \in \mathcal{B} \\
\mathbf{F}_I &= w_I - \tilde{o}_{1,I},\ \text{for } (i,j) \in \mathcal{W} \\
\mathbf{F}_I &= \left(a_I + \frac{h}{2}\mathbf{b}_I^x\right) w_{I+1} + \left(a_I - \frac{h}{2}\mathbf{b}_I^x\right) w_{I-1} + \\
&+ \left(a_I + \frac{h}{2}\mathbf{b}_I^y\right) w_{I+N-1} + \left(a_I - \frac{h}{2}\mathbf{b}_I^y\right) w_{I-N+1} + \\
&+ (h^2 c_I - 4a_I) w_I,\ \text{for } (i,j) \in \mathcal{A}
\end{aligned}$$

where we used the first order approximation to the boundary conditions, and the more accurate implementation can be achieved similarly.

We need to solve $\mathbf{F}(\mathbf{w}) = 0$, where $\mathbf{w} = (w_I), I \in \mathcal{A} \cup \mathcal{B} \cup \mathcal{W}$, a system of nonlinear equations, now written in a form suitable for the application of Newton's method. We define the Jacobian matrix $\mathbf{J}(\mathbf{w})$ as follows:

$$\begin{aligned}
\mathbf{J}_{I,I} &= 1,\ \text{for } (i,j) \in \mathcal{B} \\
\mathbf{J}_{I,I} &= 1,\ \text{for } (i,j) \in \mathcal{W} \\
\mathbf{J}_{I,I} &= h^2 \left(c_I + \frac{\partial c_I}{\partial w_I} w_I\right) - 4a_I,\ \text{for } (i,j) \in \mathcal{A}, \\
\mathbf{J}_{I,I-1} &= \left(a_I - \frac{h}{2}\mathbf{b}_I^x\right),\ \text{for } (i,j) \in \mathcal{A}, \\
\mathbf{J}_{I,I+1} &= \left(a_I + \frac{h}{2}\mathbf{b}_I^x\right),\ \text{for } (i,j) \in \mathcal{A}, \\
\mathbf{J}_{I,I-N+1} &= \left(a_I - \frac{h}{2}\mathbf{b}_I^y\right),\ \text{for } (i,j) \in \mathcal{A}, \\
\mathbf{J}_{I,I+N-1} &= \left(a_I + \frac{h}{2}\mathbf{b}_I^y\right),\ \text{for } (i,j) \in \mathcal{A}.
\end{aligned} \tag{S2-4}$$

Then, to obtain an approximation to the solution of (S2-3) using Newton's method, we start with an initial guess $\mathbf{w}^{(0)}$, and for $k = 0, 1, 2, ...$ solve

$$\mathbf{J}(\mathbf{w}^{(k)})\mathbf{X} = -\mathbf{F}(\mathbf{w}^{(k)})$$

for $\mathbf{X}$ and compute

$$\mathbf{w}^{(k+1)} = \mathbf{X} + \mathbf{w}^{(k)}.$$

To complete this section we mention that to compute o at a moment of time $t^{(\ell+1)}$ as the initial guess vector $\mathbf{w}^{(0)}$, we can use $o(t^{(\ell)})$ since it is not likely to be too far from the solution at time $t^{(\ell+1)}$. Additionally, if one wishes to let $\bar{o}$ change with time, one can set $\bar{o}(t^{(\ell+1)})$ be a function of $o(t^{(\ell)})$.

Let us point out that the diagonal terms of the Jacobian matrix are, in general, much larger in magnitude than the off-diagonal ones due to the contribution of the consumption rates c_j to the diagonal elements. As long as the Jacobian matrix is diagonally dominant it is non-singular, and therefore Newton's method converges [2].

References

1. Iserles A. A first course in the numerical analysis of differential equations. Cambridge university press; 2009.

2. Leader JJ. Numerical analysis and scientific computation. 2004.
