# Supplementary material for: Mathematical model and computational scheme for multi-phase modeling of cellular population and microenvironmental dynamics in soft tissue
Source: PLoS One. 2021 Nov 17;16(11):e0260108. doi: 10.1371/journal.pone.0260108 (PMC8598064; doi:10.1371/journal.pone.0260108)
Supplement: S3 Appendix — (PDF) [file pone.0260108.s003.pdf]

Supporting information

S3 Appendix. Numerical details, conservation of momentum and velocity fields. The key steps in the computational algorithm are the determination of the vector fields $u_j\mathbf{v}_j$ that obey the equations below.

$$\begin{aligned}&\left(\hat{\alpha}_{i0}u_i+\sum_{j=0,j\neq i}^{n+1}\hat{\alpha}_{ij}u_j\right)u_i\mathbf{v}_i+u_i\sum_{j=1,j\neq i}^{n+1}\left(\hat{\alpha}_{i0}-\hat{\alpha}_{ij}\right)u_j\mathbf{v}_j\\&=\nabla(u_i\Psi_i)-u_i\nabla\sum_{j=1}^{n+1}u_j\Psi_j,\quad i=1,...,n+1,\\&u_0\mathbf{v}_0=-\sum_{j=1}^{n+1}u_j\mathbf{v}_j,\end{aligned}\tag{S3-1}$$

and are subject to the stresses defined by

$$\Psi_i=k_i(u_0^{opt}-u_0)_+,\quad i=1,\cdots,n+1.\tag{S3-2}$$

In the simplest case we are considering here, when Ψ_i's are scalar functions (S3-2), we have, by (S3-1),

$$\begin{aligned}&\sum_{j=1}^{n+1}Q^{(i,j)}(u_j\mathbf{v}_j)^{(x)}=Z^{(i,x)},\\&\sum_{j=1}^{n+1}Q^{(i,j)}(u_j\mathbf{v}_j)^{(y)}=Z^{(i,y)},\end{aligned}\tag{S3-3}$$

for x and y components of the vectors $(u\mathbf{v})_j$, where

$$\begin{aligned}Q^{(i,j)}&=&u_i(\hat{\alpha}_{ij}-\hat{\alpha}_{i0}),\ i\neq j,\\Q^{(i,i)}&=&-(\hat{\alpha}_{i0}u_i+\sum_{j=0,j\neq i}^{n+1}\hat{\alpha}_{ij}u_j),\\Z^{(i)}&=&\nabla(u_i\Psi_i)-u_i\sum_{j=1}^{n+1}\nabla(u_j\Psi_j)\end{aligned}$$

for every $i=1,...,n+1$ at every location of the domain. It is important to note that the equations for x-components of the vectors $u_j\mathbf{v}_j$ can be separated from those for the y-components. As a result, instead of solving a system of $2K$ equations with $2K$ variables we can solve two systems of K equations with K variables, which for large systems saves computational time. (K is specified later in this section.) Another consequence of variable separation is that the $x-$ and $y-$ components of the vector fields $u_j\mathbf{v}_j$ do not have to be approximated at the same locations. More precisely, we use Eq (S3-3) to compute $u_j\mathbf{v}_j^{(x)}$ at the locations $p_{r\pm1/2,s}$ and $u_j\mathbf{v}_j^{(y)}$ at the locations $p_{r,s\pm1/2}$, $r=1,...,N-1$, $s=1,...,M-1$ only, as needed in formulas below:

$$\frac{\partial(u\mathbf{v}^{(x)})_{kj}}{\partial x}\approx\beta\frac{(u\mathbf{v}^{(x)})_{k+1/2,j}-(u\mathbf{v}^{(x)})_{k-1/2,j}}{h}+$$

$$(1-\beta)\frac{(u\mathbf{v}^{(x)})_{k+1/2,j+1/2}-(u\mathbf{v}^{(x)})_{k-1/2,j-1/2}}{2h}+$$
$$(1-\beta)\frac{(u\mathbf{v}^{(x)})_{k+1/2,j-1/2}-(u\mathbf{v}^{(x)})_{k-1/2,j+1/2}}{2h} \tag{S3-4}$$

and

$$\frac{\partial(u\mathbf{v}^{(y)})_{kj}}{\partial y}\approx\beta\frac{(u\mathbf{v}^{(y)})_{k,j+1/2}-(u\mathbf{v}^{(y)})_{k,j-1/2}}{h}+$$
$$(1-\beta)\frac{(u\mathbf{v}^{(y)})_{k+1/2,j+1/2}-(u\mathbf{v}^{(y)})_{k-1/2,j-1/2}}{2h}+$$
$$(1-\beta)\frac{-(u\mathbf{v}^{(y)})_{k+1/2,j-1/2}+(u\mathbf{v}^{(y)})_{k-1/2,j+1/2}}{2h}, \tag{S3-5}$$

For the additional locations we simply use averages of the computed values. For example

$$(u\mathbf{v})^{(x)}_{r+1/2,s+1/2}=\frac{(u\mathbf{v})^{(x)}_{r+1/2,s}+(u\mathbf{v})^{(x)}_{r+1/2,s+1}}{2}$$

and

$$(u\mathbf{v})^{(y)}_{r+1/2,s+1/2}=\frac{(u\mathbf{v})^{(y)}_{r,s+1/2}+(u\mathbf{v})^{(y)}_{r+1,s+1/2}}{2}.$$

Note that the matrices Q and the right-hand-side arrays Z in (S3-3) are needed at locations $p_{r-1/2,s},\ r=1,...,N,\ s=1,...,M-1$ for the x-component system of equations and at the locations $p_{r,s-1/2},\ r=1,...,N-1,\ s=1,...,M$ for the y-components. The quantities defining Q and Z are known and computed at locations $p_{r,s}$. At the half-locations we use averages to approximate the required values, that is

$$u_{r+1/2,s}=\frac{u_{r,s}+u_{r+1,s}}{2}$$

and

$$u_{r,s+1/2}=\frac{u_{r,s}+u_{r,s+1}}{2}.$$

If a half-location belongs to the boundary of Ω we use the quantities' boundary values. Finally, the gradients in Z are approximated by

$$\frac{\partial(u\Psi)}{\partial x}(p_{r-1/2,s})\approx\frac{u_{r,s}\Psi_{r,s}-u_{r-1,s}\Psi_{r-1,s}}{h}$$

and

$$\frac{\partial(u\Psi)}{\partial y}(p_{r,s-1/2})\approx\frac{u_{r,s}\Psi_{r,s}-u_{r,s-1}\Psi_{r,s-1}}{h},$$

for the locations interior to Ω. When half-point locations belong to the boundary of Ω we use one-sided formulas.

When the system of Eq (S3-3) is encoded, it is convenient to group the variables according to their position in space. More specifically, let $I=1,...,L$ be the index identifying a location on the grid $p_{r,s}$, and let i,j be indices identifying subpopulations of cells or the ECM. Denote $Q^{(i,j)},\ i,j=1,...,n+1$ evaluated at p_I by $Q^{(i,j)}_I$, let $(u\mathbf{v})^{(x)}_j$ evaluated at p_I be denoted by $(u\mathbf{v})^{(x)}_{I,j}$, and let $Z^{(i,x)}$ evaluated at p_I be denoted by $Z^{(i,x)}_I$ and similarly for the y components.

Next, let $\mathbf{w}^{(x)}$ denote a column vector consisting of $(u\mathbf{v})^{(x)}_{I,j}$ for all the subpopulations $j=1,..,n+1$ at all the locations $p_I,\ I=1,...,L$ ordered as follows:

$$\mathbf{w}^{(x)}=((u\mathbf{v})^{(x)}_{1,1},(u\mathbf{v})^{(x)}_{1,2},...,(u\mathbf{v})^{(x)}_{1,n+1},(u\mathbf{v})^{(x)}_{2,1},(u\mathbf{v})^{(x)}_{2,2},...,(u\mathbf{v})^{(x)}_{2,n+1},...(u\mathbf{v})^{(x)}_{L,n+1})^T.$$

Define a matrix of coefficients A as a block-diagonal matrix with blocks $Q_I^{(i,j)}$, ordered to correspond to $\mathbf{w}^{(x)}$, along the main diagonal, that is

$$A = \begin{pmatrix} Q_1^{(1,1)} & Q_1^{(1,2)} & ... & Q_1^{(1,n+1)} & ... & ... & ... & ... & ... \\ Q_1^{(2,1)} & Q_1^{(2,2)} & ... & Q_1^{(2,n+1)} & ... & ... & ... & ... & ... \\ ... & ... & ... & ... & ... & ... & ... & ... & ... \\ Q_1^{(n+1,1)} & Q_1^{(n+1,2)} & ... & Q_1^{(n+1,n+1)} & ... & ... & ... & ... & ... \\ ... & ... & ... & ... & ... & ... & ... & ... & ... \\ ... & ... & ... & ... & ... & Q_L^{(1,1)} & Q_L^{(1,2)} & ... & Q_L^{(1,n+1)} \\ ... & ... & ... & ... & ... & Q_L^{(2,1)} & Q_L^{(2,2)} & ... & Q_L^{(2,n+1)} \\ ... & ... & ... & ... & ... & ... & ... & ... & ... \\ ... & ... & ... & ... & ... & Q_L^{(n+1,1)} & Q_L^{(n+1,2)} & ... & Q_L^{(n+1,n+1)} \end{pmatrix}.$$

Define a column vector $\mathbf{B}^{(x)}$ as

$$\mathbf{B}^{(x)} = (Z_{1,1}^{(x)}, Z_{1,2}^{(x)}, ..., Z_{1,n+1}^{(x)}, ..., Z_{L,1}^{(x)}, Z_{L,2}^{(x)}, ..., Z_{L,n+1}^{(x)})^T$$

ordered similarly to $\mathbf{w}^{(x)}$. We solve the system $A\mathbf{w}^{(x)} = \mathbf{B}^{(x)}$ and set up and solve a similar system for $\mathbf{w}^{(y)}$.

The $Q_I^{(i,j)},\ i,j = 1,...,n+1$ blocks of matrix A are $(n+1)\times(n+1)$ square matrices varying from location to location. We have a simple sufficient condition for these blocks to be invertible.

Theorem. Suppose the drag coefficients $\hat{\alpha}_{ij} \neq 0$ for all $i \neq j$, and satisfy

$$\hat{\alpha}_{i0} > \sum_{j=1, j\neq i}^{n+1} |\hat{\alpha}_{ij} - \hat{\alpha}_{i0}|$$

for every $i = 1, ..., n+1$. Then $Q_I^{(i,j)}$ $i,j = 1,...,n+1$ is invertible at every location p_I of the domain.

Proof. If $u_i = 0$ at at some location p_I in the domain, then the i-th row of $Q_I^{(i,j)}$ has only one non-zero entry. It is located on the main diagonal and is equal to

$$-\sum_{j=0, j\neq i}^{n+1} \hat{\alpha}_{ij} u_j.$$

This value is non-zero, since $u_i = 0$ and $\sum_{j=0}^{n+1} u_j = 1$ imply that there is at least one non-zero volume fraction at the same location.

If u_i is not zero at p_I, then it follows from the condition above that the diagonal entry of $Q_I^{(i,j)}$

$$-(\hat{\alpha}_{i0} u_i + \sum_{j=0, j\neq i}^{n+1} \hat{\alpha}_{ij} u_j)$$

has an absolute value

$$\hat{\alpha}_{i0} u_i + \sum_{j=0, j\neq i}^{n+1} \hat{\alpha}_{ij} u_j \geq \hat{\alpha}_{i0} u_i > u_i \sum_{j=1, j\neq i}^{n+1} |\hat{\alpha}_{ij} - \hat{\alpha}_{i0}|.$$

Therefore $Q_I^{(i,j)}$ is strictly diagonally dominant and invertible.

It follows that A is invertible as well, and thus the two systems of equations for determining $u\mathbf{v}$ both have unique solutions, as long as the conditions on the drag coefficients are satisfied.

These conditions can be reinterpreted in the following way. Note that

$$\frac{|\hat{\alpha}_{i0} - \hat{\alpha}_{ij}|}{\hat{\alpha}_{i0}}$$

is a relative difference between the drag coefficients $\hat{\alpha}_{ij}$ and $\hat{\alpha}_{i0}$. As long as the sum over $j = 1, ..., n+1,\ j \neq i$ of these relative differences is bounded by 1 (i.e., the drag coefficients $\hat{\alpha}_{ij}$ are not very different from $\hat{\alpha}_{i0}$), the condition above is satisfied, and the system has a unique solution. For a typical application that we are interested in $n \leq 4$. If we keep the relative differences between the drag coefficients less than 25%, the systems will always be solvable.
