# Supplementary material for: Mathematical model and computational scheme for multi-phase modeling of cellular population and microenvironmental dynamics in soft tissue
Source: PLoS One. 2021 Nov 17;16(11):e0260108. doi: 10.1371/journal.pone.0260108 (PMC8598064; doi:10.1371/journal.pone.0260108)
Supplement: S4 Appendix — (PDF) [file pone.0260108.s004.pdf]

Supporting information

S4 Appendix. Stability Considerations and Time Step There are natural constraints on the time step dictated by the understanding that all of the components have nonnegative volume fractions bounded by unity. In fact, the sum of all volume fraction excluding the interstitial fluid is bounded above by one. Assuming that this condition holds at the time step t_ℓ, we must ensure that it also holds at the time step $t_{\ell+1}$.

It is clear that if components in the model grow too fast or move too fast over the given time interval Δt, our numerical prediction for the component values at time $t_{\ell+1}$ may lead to unacceptable values. The time step Δt need to be kept small enough to avoid such overshootings. Below is the estimate on the time step meant to ensure the stability of the computational process.

Consider the 4th step in Algorithm 1 applied to the i-th component:

$$u_i^{\ell+1} = u_i^\ell + \Delta t \left(g_i^\ell - \nabla \cdot (u_i \mathbf{v}_i)^\ell \right).$$

Sum over $i = 0, ..., n+1$, and note that the condition

$$\Delta t \leq \frac{1}{|\sum_{i=1}^{n+1} g_i^\ell| + |\sum_{i=1}^{n+1} \nabla \cdot (u_i \mathbf{v}_i)^\ell|}, \ell = 1, 2, ..., \tag{S4-1}$$

sufficiently controls the time step size. Since growth and decay functions defining g_i's and $u_i \mathbf{v}_i$'s depend on constitutive stress-strain relations specific to a problem at hand, further stability considerations are discussed tied to the specifics of the problem at hand. Below we establish the time step constraints for the tissue regeneration examples described in Simulations and Experimental Results.

For the tissue regeneration simulations we can satisfy the stability condition (S4-1) by requiring that

$$\Delta t \leq \frac{1}{|b_1| + |b_2| + |\nabla \cdot (u_1 \mathbf{v}_1) + \nabla \cdot (u_2 \mathbf{v}_2)|}$$

at all times. This condition can be satisfied by requiring that

$$\Delta t \leq \frac{1}{b_{1,\max} u_0 \|\nabla [(u_0 - u_0^{opt})_+]\|_2 + b_{2,\max} u_0 u_1 (u_2^{opt} - u_2)_+ + |\nabla \cdot (u_1 \mathbf{v}_1) + \nabla \cdot (u_2 \mathbf{v}_2)|}$$

at all times. This last condition can be simplified further as

$$\Delta t \leq \frac{1}{b_{1,\max}(1 - u_0^{opt})/h + b_{2,\max} u_2^{opt} + |\nabla \cdot (u_1 \mathbf{v}_1) + \nabla \cdot (u_2 \mathbf{v}_2)|}.$$

For our next step consider the estimates for $\nabla \cdot (u_i \mathbf{v}_i), \quad i = 1,\ 2$. When drag coefficients are all the same, which in the experiments presented here they are, we can express

$$u_1 \mathbf{v}_1 = \frac{1}{\hat{\alpha}_{10} u_1 + \hat{\alpha}_{10} u_0 + \hat{\alpha}_{12} u_2} ((1 - u_1) \nabla (u_1 \Psi_1) - u_1 \nabla (u_2 \Psi_2)).$$

Note that

$$\left| \frac{\partial}{\partial x} (u_i (u_0^{opt} - u_0)_+) \right| \leq u_0^{opt}/h, \quad i = 1, 2$$

and similarly for the other component of the gradient, and thus

$$\left|(1-u_1)\frac{\partial}{\partial x}(u_1\Psi_1) - u_1\frac{\partial}{\partial x}(u_2\Psi_2)\right| \leq (k_1+k_2)u_0^{opt}/h. \tag{S4-2}$$

Then

$$|u_1\mathbf{v}_1^x| \leq \frac{1}{\hat{\alpha}}(k_1+k_2)u_0^{opt}/h$$

and similarly for its y-component and $|u_2\mathbf{v}_2|$. Therefore.

$$|\nabla\cdot(u_1\mathbf{v}_1)+\nabla\cdot(u_2\mathbf{v}_2)| \leq \frac{4}{\hat{\alpha}}(k_1+k_2)u_0^{opt}/h^2,$$

and the condition

$$\Delta t \leq \frac{1}{b_{1,\max}(1-u_0^{opt})/h + b_{2,\max}u_2^{opt} + \frac{4}{\hat{\alpha}}(k_1+k_2)u_0^{opt}/h^2}$$

ensures stability. Naturally, since we assumed the worst case scenario throughout the process, in practice a larger time step may suffice.
